# Mechanically robust bamboo node and its hierarchically fibrous structural design

**DOI:** 10.1093/nsr/nwac195

**Published:** 2022-09-22

**Authors:** Si-Ming Chen, Si-Chao Zhang, Huai-Ling Gao, Quan Wang, LiChuan Zhou, Hao-Yu Zhao, Xin-Yu Li, Ming Gong, Xiao-Feng Pan, Chen Cui, Ze-Yu Wang, YongLiang Zhang, HengAn Wu, Shu-Hong Yu

**Affiliations:** Department of Chemistry, Institute of Biomimetic Materials & Chemistry, Anhui Engineering Laboratory of Biomimetic Materials, Division of Nanomaterials & Chemistry, Hefei National Research Center for Physical Sciences at the Microscale, Institute of Energy, Hefei Comprehensive National Science Center, University of Science and Technology of China, Hefei 230026, China; Department of Chemistry, Institute of Biomimetic Materials & Chemistry, Anhui Engineering Laboratory of Biomimetic Materials, Division of Nanomaterials & Chemistry, Hefei National Research Center for Physical Sciences at the Microscale, Institute of Energy, Hefei Comprehensive National Science Center, University of Science and Technology of China, Hefei 230026, China; Department of Chemistry, Institute of Biomimetic Materials & Chemistry, Anhui Engineering Laboratory of Biomimetic Materials, Division of Nanomaterials & Chemistry, Hefei National Research Center for Physical Sciences at the Microscale, Institute of Energy, Hefei Comprehensive National Science Center, University of Science and Technology of China, Hefei 230026, China; CAS Key Laboratory of Mechanical Behavior and Design of Materials, Department of Modern Mechanics, Engineering and Materials Science Experiment Center, University of Science and Technology of China, Hefei 230027, China; CAS Key Laboratory of Mechanical Behavior and Design of Materials, Department of Modern Mechanics, Engineering and Materials Science Experiment Center, University of Science and Technology of China, Hefei 230027, China; Department of Chemistry, Institute of Biomimetic Materials & Chemistry, Anhui Engineering Laboratory of Biomimetic Materials, Division of Nanomaterials & Chemistry, Hefei National Research Center for Physical Sciences at the Microscale, Institute of Energy, Hefei Comprehensive National Science Center, University of Science and Technology of China, Hefei 230026, China; Department of Chemistry, Institute of Biomimetic Materials & Chemistry, Anhui Engineering Laboratory of Biomimetic Materials, Division of Nanomaterials & Chemistry, Hefei National Research Center for Physical Sciences at the Microscale, Institute of Energy, Hefei Comprehensive National Science Center, University of Science and Technology of China, Hefei 230026, China; CAS Key Laboratory of Mechanical Behavior and Design of Materials, Department of Modern Mechanics, Engineering and Materials Science Experiment Center, University of Science and Technology of China, Hefei 230027, China; Department of Chemistry, Institute of Biomimetic Materials & Chemistry, Anhui Engineering Laboratory of Biomimetic Materials, Division of Nanomaterials & Chemistry, Hefei National Research Center for Physical Sciences at the Microscale, Institute of Energy, Hefei Comprehensive National Science Center, University of Science and Technology of China, Hefei 230026, China; Department of Chemistry, Institute of Biomimetic Materials & Chemistry, Anhui Engineering Laboratory of Biomimetic Materials, Division of Nanomaterials & Chemistry, Hefei National Research Center for Physical Sciences at the Microscale, Institute of Energy, Hefei Comprehensive National Science Center, University of Science and Technology of China, Hefei 230026, China; Department of Chemistry, Institute of Biomimetic Materials & Chemistry, Anhui Engineering Laboratory of Biomimetic Materials, Division of Nanomaterials & Chemistry, Hefei National Research Center for Physical Sciences at the Microscale, Institute of Energy, Hefei Comprehensive National Science Center, University of Science and Technology of China, Hefei 230026, China; CAS Key Laboratory of Mechanical Behavior and Design of Materials, Department of Modern Mechanics, Engineering and Materials Science Experiment Center, University of Science and Technology of China, Hefei 230027, China; CAS Key Laboratory of Mechanical Behavior and Design of Materials, Department of Modern Mechanics, Engineering and Materials Science Experiment Center, University of Science and Technology of China, Hefei 230027, China; Department of Chemistry, Institute of Biomimetic Materials & Chemistry, Anhui Engineering Laboratory of Biomimetic Materials, Division of Nanomaterials & Chemistry, Hefei National Research Center for Physical Sciences at the Microscale, Institute of Energy, Hefei Comprehensive National Science Center, University of Science and Technology of China, Hefei 230026, China; Institute of Innovative Materials (I2M), Department of Materials Science and Engineering, Department of Chemistry, Southern University of Science and Technology, Shenzhen 518055, China

**Keywords:** biological materials, bamboo node, hierarchical fiber, structural reinforcement, functional integration

## Abstract

Although short bamboo nodes function in mechanical support and fluid exchange for bamboo survival, their structures are not fully understood compared to unidirectional fibrous internodes. Here, we identify the spatial heterostructure of the bamboo node via multiscale imaging strategies and investigate its mechanical properties by multimodal mechanical tests. We find three kinds of hierarchical fiber reinforcement schemes that originate from the bamboo node, including spatially tightened interlocking, triaxial interconnected scaffolding and isotropic intertwining. These reinforcement schemes, built on porous vascular bundles, microfibers and more-refined twist-aligned nanofibers, govern the structural stability of the bamboo via hierarchical toughening. In addition, the spatial liquid transport associated with these multiscale fibers within the bamboo node is experimentally verified, which gives perceptible evidence for life-indispensable multidirectional fluid exchange. The functional integration of mechanical reinforcement and liquid transport reflects the fact that the bamboo node has opted for elaborate structural optimization rather than ingredient richness. This study will advance our understanding of biological materials and provide insight into the design of fiber-reinforced structures and biomass utilization.

## Teaser

Mechanical reinforcement schemes and underlying mechanisms within the sophisticated multiscale fibrous structure of one bamboo node have been discovered, providing insight into engineering structural material design and biomass utilization.

## INTRODUCTION

**Figure 1.**
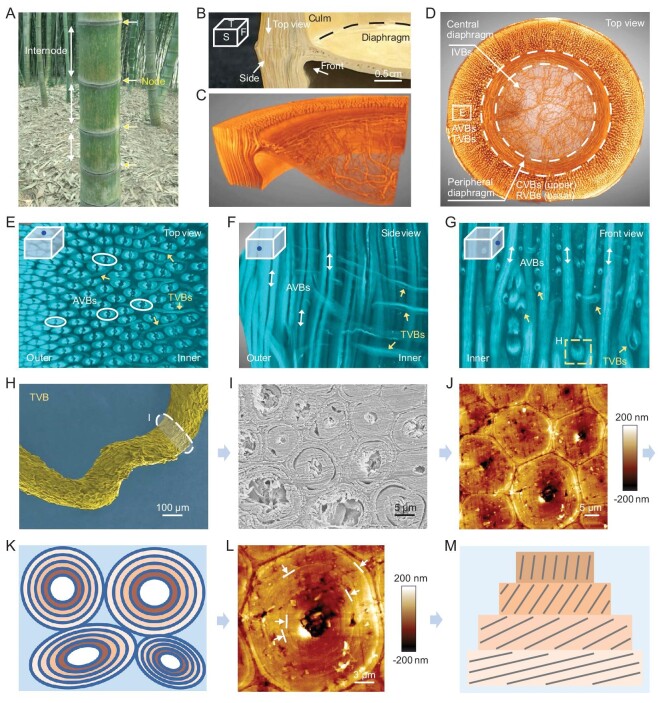
Morphology and hierarchical fiber arrangement of the bamboo node. (A) Digital image of moso bamboos (*Phyllostachys edulis*). Yellow arrows and white arrows show nodes and internodes of one bamboo, respectively. (B) Partial node showing pre-defined view directions (abbreviations: T, top view; S, side view; F, front view). (C and D) Reconstructed 3D configuration of partial and entire nodes highlighting fibrous VBs. AVBs and TVBs exist at the node culm, CVBs and RVBs exist at the peripheral diaphragm, and isotropic IVBs exist at the central diaphragm. (E–G) Snapshots of reconstructed 3D configurations of the node culm, showing complicated interweaving of AVBs and TVBs. Some TVBs bifurcate (E). Some AVBs twist due to the entwining and squeezing caused by TVBs (G). (H) Scanning electron microscope (SEM) image of one detached TVB showing twisted state. The white marker indicates its cross section. (I and J) Cross-section SEM and atomic force microscope (AFM) images of one twisted TVB that is composed of many multilayered microfibers. (K) Cross-section schematic of one twisted TVB. (L) AFM image focusing on one multilayered microfiber. Arrows and lines indicate multilayers. (M) Schematic of one microfiber that is composed of many twist-aligned nanofiber lamellae. Black lines represent aligned nanofibers, different yellow boxes represent different nanofiber lamellae.

Nature creates many fascinating biological materials, including lightweight bamboo, tough nacre, fatigue-resistant bone, adhesive mussel byssal thread, water-repellent strider legs and so on [1–4]. As one sustainable resource featuring superior mechanical properties and rapid growth, moso bamboo (*Phyllostachys edulis*) has an extremely long hollow stalk (internode) that is segmented by many nodes. These nodes are densely distributed at the root and top and loosely distributed at the middle (Fig. S1 in the Supplementary Data). Besides parenchyma cells (PCs), vascular bundles (VBs) that consist of fibrous sheath, xylem and phloem are the main building blocks of bamboo (Fig. S2) [5]. Possessing axial VBs (AVBs) with radial-gradient distribution (Figs S2 and S3), the segmented tubular internodes occupy bamboo's main body (Fig. 1A)and Fig. S1A) and play a key role in mechanically supporting body growth [5–7]. This natural unidirectional structure of the internode, like that of synthetic fibrous composites assembled from carbon nanotubes and carbon or Kevlar fibers, has attracted extensive attention [7,8]. The excellent mechanical property, cost-effectiveness, eco-friendliness and sustainability of the internode can meet stringent engineering requirements, making bamboo a promising resource in the manufacture of structural materials for wide applications such as house furniture, engineering construction and wind turbine blades [5–7]. However, compared to the internode, the bamboo node seems to be mechanically weak for engineering applications and thus is often deserted during processing [6,7,9]. The act of artificially discarding nodes overlooks the ingenious design of biological materials over the long-term evolutionary process. For bamboo survival, the short node is also key; it functions as lateral fluid exchange and fixed-point mechanical reinforcement [10–12]. This multifunctional realization should be closely related to the node's structure and implies natural intelligence [1–4,13–16].

In the past, efforts have been made to explore the structure and properties of the node [11,17–21]. Compared to the tubular internode, the node is thick and contains a diaphragm (or termed septa) [11,17–20]. The microstructure of the node was studied. It was generally accepted that some AVBs from the internode deflect and thicken at the node culm, and some deflected VBs enter the diaphragm through elusive movement trails [18,20]. Further, the relationship between the unevenly spaced distribution of nodes along the stalk and the structural stability of the bamboo was investigated [17]. Theoretical work showed that the node diaphragm can be considered as a homogeneous spring-like joint that can store and release strain energy to maintain structural stability under external loading [11]. Mechanical experiments showed the differences in macroscale properties between the node and the internode [18–21], partly verifying the node's positive effect on restraining stalk buckling [21]. It is worth noting that the spatial hierarchy of VBs within the node and its detailed contribution to mechanical reinforcement and liquid transport were not sufficiently researched until now. Revisiting bamboo nodes is a worthy endeavor, which will inspire engineering structural design.

Herein, we systematically study the hierarchical heterostructure of the bamboo node and its rationality via both experiments and simulations. Multiscale imaging techniques (that utilize signals of light, X-rays, electron, and force), multimodal mechanical tests, *in-situ* characterization, channel and liquid transport detection, and finite element analysis are combined. Several fiber reinforcement schemes and the underlying mechanisms are demonstrated for the first time, highlighting some key fibrous VBs that contribute to the structural stability of bamboo. Further, the microfiber bundles and more-refined twist-aligned nanofibers [1] that make up VBs are expected to play important roles in multiscale strengthening and toughening via their slippage and pull-out. In addition, we substantiate the multidirectional liquid transport path of the node culm and demonstrate the node's integrated functions of mechanical reinforcement and liquid transport via a solar thermal water evaporation device. These findings will deepen our understanding of biological materials, including nodes, and promote reasonable structural design of fibrous engineering materials and applications of nodes.

## RESULTS AND DISCUSSION

### Hierarchical organization of the bamboo node

A bamboo node contains a node culm and diaphragm (Fig. 1A)and B). Due to the existence of AVBs, the node culm inherits the internode's structural features to some extent. AVBs within the node culm are assembled by microfibers (Figs S4 and S5) [22,23]. These microfibers are found to exhibit position-dependent compactness differences. For the inner AVBs, the peripheral microfibers display micropores and sublayers, while the central microfibers appear compact (Fig. S6). For the outer AVBs, all the microfibers display dense arrangements without pores (Fig. S7). Vickers hardness measurements along the radial direction reflect the tendency to be rigid on the outside and soft on the inside (Fig. S8). Not limited to surface observation and measurement, microfibers can be separated through chemical dissociation for more extensive study. Statistical analysis shows that the length of the outer microfibers is larger than that of the inner microfibers, but their diameter is smaller (Fig. S9). The densely distributed AVBs composed of long and thin microfibers at the outer culm found here are expected to directly resist external loads [1,24,25]. It can be speculated that there is a ‘rigid outer culm to soft inner culm’ transition and a ‘porous PCs to porous peripheral microfibers to dense central microfibers’ transition (for the inner culm). These structural features are expected to consecutively facilitate stress transfer and strain energy dissipation of the node culm.

**Figure 2.**
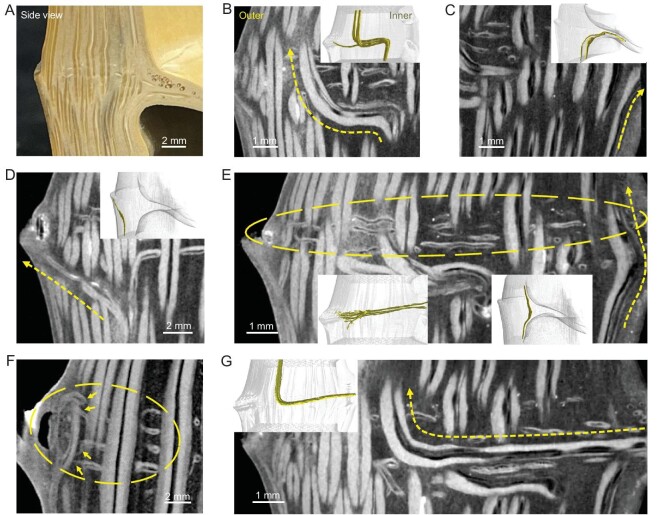
Fibrous arrangement of the node culm. (A) Digital image of the node culm. (B–D) Micro-CT images show that AVB (B) transversally deflects and continues to grow upward, (C) transversally deflects into the diaphragm and (D) to the outside, and (E right, and right inset) bends inward and thickens. Arrows (B–E) indicate the VBs’ arrangement. All insets are reconstructed 3D configurations of the node culm highlighting VBs. (E and F) Micro-CT images show that small, thin and high-positioned TVBs link the diaphragm and outside. Left inset (E), circles (E and F) and arrows (F) show the connection between these thin TVBs and outside. (G) Micro-CT images show that VB emerges from the diaphragm and continues to grow upward. Note that there are many channels within the VBs, appearing as black lines between fibrous bundles (white zone).

Not limited to the features contributed by AVBs, the node also has transversal VBs (TVBs) (interlaced with AVBs) within the node culm and a VB-assembled diaphragm (Fig. 1B–G and Movies S1–S5). The micro-computerized tomography (micro-CT) technique provides a non-destructive method for studying these features [26]. As shown in Fig. 1G–M and Movies S2 and S3, the TVBs are twisted and composed of layered microfibers that possess twist-aligned nanofibers [1], and the hierarchy of the fibrous structure is fully reflected here. In fact, TVBs are not uniform in diameter (Fig. 1G), probably hinting at their different origins. Because they exist only at the node culm but not the internode, TVBs are likely to evolve from AVBs or be associated with the diaphragm. Based on this consideration, we paid attention to the subtle changes in the AVBs around the node. Using the micro-CT technique, we found that the AVBs deflect and thicken at the node (Fig. 2 and Movie S4). Surprisingly, and different from a previous report [20], the deflection found here is multiple: some AVBs transversally deflect for some distance and continue to extend upward (Fig. 2B); some AVBs deflect into the diaphragm (Fig. 2C) or to the outside (lateral branch or sheath or ridge) (Fig. 2D); and some AVBs slightly bend inward and thicken at the inner culm (Fig. 2E)(right)). These spatial variations of AVBs partly explain the origins of TVBs. Unlike those with axial deflection, we further found some small, thin and high-positioned VBs that link the diaphragm and the outside (Fig. 2E)(left) and F, and Movie S4), and found some VBs that come out from the diaphragm and extend upward (Fig. 2G). These two types of VBs further explain the origins of TVBs. Based on the top view of the node culm slices (Fig. S10A–C), we confirmed that all TVBs travel in a twisted manner and some thin TVBs bifurcate. Due to the possible squeezing between TVBs and AVBs, AVBs also exhibit twisted states (Fig. 1G, Fig. S10D and Movie S3). It can be speculated that these twisted and bifurcated VBs create tightly interlocked structures surrounded by PCs (Fig. 1G, Fig. S10, Movies S3 and S5). The microfibers and twist-aligned nanofibers that make up the twisted VBs within the interlocked organization will promote energy absorption for structural stability through the motions of hierarchical fibers [22,23].

**Figure 3.**
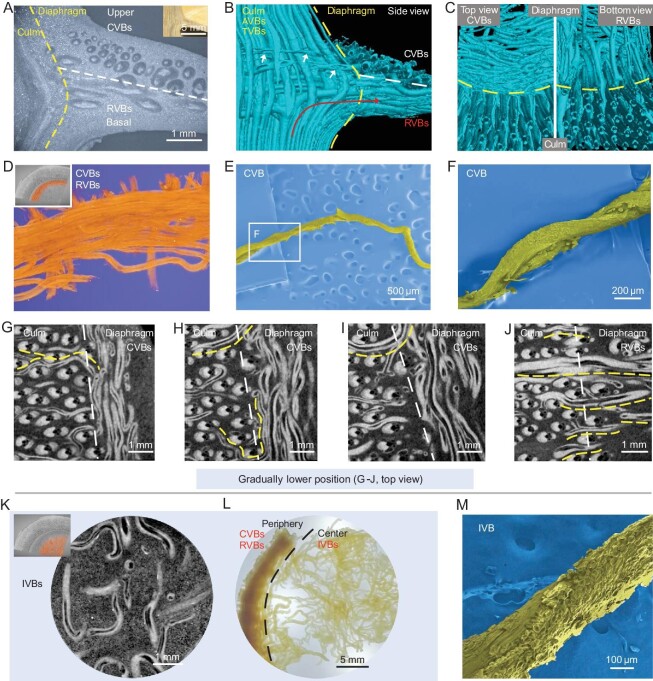
Fibrous arrangement of the diaphragm. (A) Optical microscope (OM) image of one peripheral diaphragm (culm–diaphragm transition zone, side view), showing position-dependent partitions of three different fibrous zones. Inset is one real specimen. (B and C) Reconstructed 3D configurations of one peripheral diaphragm from different view directions, showing the spatially triaxial perpendicular arrangement of VBs. These position- and thickness-dependent fibrous VBs can be divided into two categories, (C, right) the RVBs at the basal periphery and (C, left) the small and thin CVBs at the upper periphery. RVB comes from deflected AVB (B, indicated by red arrow), and CVBs connect to the outside (B, indicated by white arrows). (D) False-colored peripheral diaphragm showing CVBs (top view). (E and F) SEM images of one CVB showing twisted state. (G–J) Slice projections of the ‘node culm–diaphragm’ transition zone showing position-dependent CVBs (G-I, top) and RVBs (J, bottom) at the peripheral diaphragm. The white lines (G–J) distinguish the position of the node culm and diaphragm. Thin, high-positioned TVBs from the node culm link the diaphragm, resulting in circumferential arrangement (indicated by yellow lines (G–I)). Low-positioned TVBs from the node culm link the diaphragm, resulting in radial arrangement (indicated by yellow lines (J)). (K) Slice projection of the central diaphragm showing porous IVBs (top view). Inset is the sampling site (false color). (L) Digital image of one delignified diaphragm (top view), showing dense periphery (CVBs and RVBs) and loosened IVBs at the central diaphragm. (M) SEM image of one detached IVB, showing twisted state.

**Figure 4.**
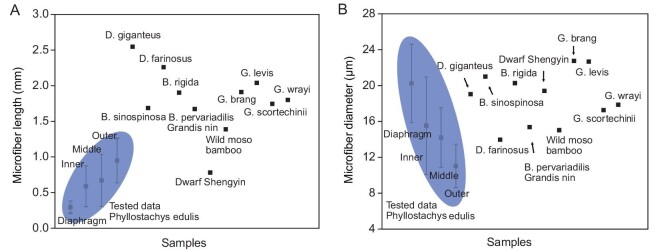
Comparison between the (A) microfiber length and (B) diameter obtained in this work and the data reported in the literature. More detailed data are listed in Tables S1 and S2 in the Supplementary Data.

The diaphragm is another unique feature of the node, and is thick at the periphery and thin at the center (Fig. 2D)inset). It is much softer than the culm (Fig. S11) and is like an embedded concave plate (Fig. 1B)and C). It can be considered as one VB-assembled heterogeneous composite (Fig. 1D)and Movie S1) and distinguished into three parts: the circumferential VBs (CVBs) zone at the upper periphery (Fig. 3A–I and Movie S6), the radial VBs (RVBs) zone at the basal periphery (Fig. 3A–C, J and Movie S6) and the isotropic intertwined VBs (IVBs) zone at the central diaphragm (Fig. 3K). Delignification dissociation further reflects the position-dependent difference of the diaphragm, highlighting the dense periphery and the loosened central IVBs (Fig. 3L). For the upper periphery, the twisted CVBs (Fig. 3B–F) connect to the outside (Fig. 2E)and F, Fig. S10A and B, and Movie S4), and inwardly enter the central diaphragm with disordered states after surrounding the inner culm wall for some distance. Instead of forming a continuous closed loop, these CVBs are discontinuous (Fig. 3G–I) like the discrete aragonite microplates of natural nacre [1]. For the basal periphery, the RVBs outwardly connect to the AVBs that deflect inward (Figs 2C)and 3B, C and J), instead of forming a straightforward radial structure (central radiation structure). They also become disordered after inward radial walking for some distance. The isotropically distributed and twisted IVBs at the central diaphragm (Fig. 3K–M) naturally evolve from the CVBs and RVBs at the periphery. It is worth noting that, for the diaphragm, we only consider the traveling of VBs from the outside to the center rather than the reverse (see Fig. 2G), because the traveling direction does not affect the entire structure presentation. All these structural features are recorded in Movie S7, which slices from the diaphragm to the culm. On a much finer scale, we obtained microfibers that make up VBs within the diaphragm. Compared to the microfibers from culms (Fig. S9), microfibers here are short, thick and slightly twisted (Fig. S12). This unique microfiber morphology may be related to the fact that the diaphragm is used to store and release strain energy [11] but is not directly subjected to external loading, because the general understanding is that long fibers can more effectively withstand tension, bending and other loads [6,7]. Overall, the length and diameter of the microfibers within the bamboo (diaphragm and culm) are comparable to those of various bamboos previously reported (Fig. 4, Tables S1 and S2). Interestingly, the hierarchical fibrous arrangement of the diaphragm bears a resemblance to that of the human meniscus [27,28]. The meniscus contains radially aligned collagen fiber bundles at the periphery and circumferentially arranged collagen fiber bundles and intertwined micro-zones. These organized collagen fiber bundles give bone excellent weight-bearing and anti-vibration abilities [27,28]. The ‘node culm–diaphragm’ transition zone (peripheral diaphragm) particularly attracts our attention (Fig. 3A)and B), because it is a bridge for outside-in stress transfer and probably has inspiration for the widely concerned engineering heterogeneous connection. The triaxial mutually perpendicular fibrous organization (X, RVBs at the basal periphery; Y, CVBs at the upper periphery; Z, AVBs at the inner culm) is its remarkable feature (Fig. 3A–C, G–J and Movie S6). These VBs seem to form a scaffolding structure for connection. Unlike the engineering scaffolding, which uses extrinsic fasteners, the natural scaffolding found here, consisting of spatially interlaced hierarchical fibers, is organized and holistic with greater structural advantage. It is worth noting that this unique scaffolding structure is a common feature of nodes at the root, middle and top parts of one bamboo (Fig. S13), probably hinting at high-level structural design principles.

As far as we know, this is the first time that the sophisticated fibrous interconnectivity and structure of the bamboo node have been revealed. Here, for the node, we speculate that there are three kinds of reinforcement schemes that contribute to the structural stability of bamboo: the tightly interlocked structure (by AVBs-TVBs) of the node culm (Fig. 1E–G and Movies S3–S5), the scaffolding structure (by RVBs-CVBs-AVBs) of the ‘node culm–diaphragm’ transition zone (Fig. 3A–C and Movie S6) and the isotropic intertwined structure (by IVBs) of the central diaphragm (Figs 1D)and 3K)and L). These schemes are spatially interconnected and form one integrated microsystem. To understand their structure–properties relationship, several simplified models were constructed. As shown in Fig. S14A, under compression load, the transversal fiber changes the deformation of the axial fiber by ‘blocking effect’. The ‘blocking effect’ can slow down performance degradation (Fig. S14B). Although the fibrous hierarchy (nanofiber-to-microfiber), twisted state and tight interlocking of AVBs-TVBs are not considered (Fig. 1E–M), the simulated fibrous combination can represent the basic structural feature of the node culm. It can be speculated that TVBs and derived interlocked structures (Fig. 1E–M) can act as mechanical reinforcement. Further, for the ‘node culm–diaphragm’ transition zone, it demonstrates that the discontinuous CVBs at the peripheral diaphragm are more conducive to resisting external loads, which improves the stiffness of the node (Fig. S15). Like the motions of the discontinuous aragonite microplates within nacre to toughen [1,14], the motions of the discontinuous CVBs are beneficial for mechanical properties. In addition, the combination of the RVBs at the periphery and the soft isotropic IVBs at the center seems to be flexible and can improve strength and toughness to avoid premature failure, compared to the central radially structural scenario (Fig. S16). All these models demonstrate the node's structural superiority. Note that the fibrous hierarchy will contribute to the structural stability of the node and bamboo in the natural world [1]. Frankly, the cross-scale simulations of biological hierarchical fiber structures are still tough here.

### Mechanical investigation of the node culm and diaphragm

**Figure 5.**
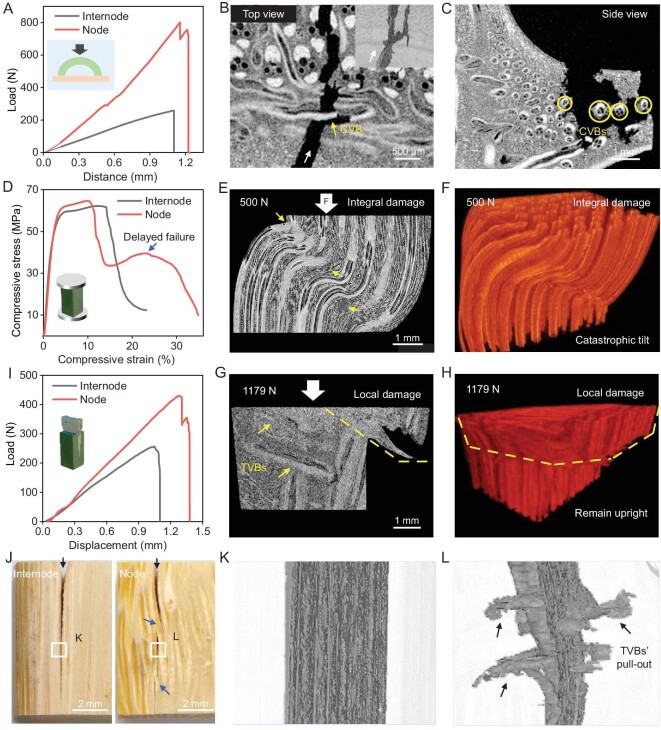
Mechanical studies of CVBs at the peripheral diaphragm and TVBs at the node culm. (A) Compression load-displacement curves of the internode and node-culm-related arched specimens. Inset shows a sketch map. (B and C) Slice projections of the node culm specimen after compression from different view directions. The white arrow (B) shows crack direction, the yellow arrow (B) shows CVB-induced crack deflection. Inset is the reconstructed 3D crack, showing that CVBs can direct crack deflection and bifurcation. Yellow circles (C) indicate the cross sections of CVBs and show their pull-out or fracture. (D) Compression stress-strain curves of the internode culm and node culm, showing delayed failure of the latter. Inset shows a sketch map. (E and F) Slice projection and reconstructed 3D structure of the internode under *in-situ* compression, showing integral damage. Most AVBs were fractured and PCs were torn (indicated by arrows). (G and H) Slice projection and reconstructed 3D structure of the node culm under *in-situ* compression. They indicate that the presence of TVBs (indicated by arrows) changes stress transfer mode and enables local damage rather than integral damage. (I) Splitting load-displacement curves of the internode culm and node culm. Inset shows a sketch map. (J) Digital images of the internode culm and node culm after splitting, showing macroscopic cracks. Black arrows show crack directions. Twisted and bridged cracks exist in the node (blue arrows), while a straight crack exists in the internode. (K and L) Reconstructed 3D cracks of the internode culm and node culm after splitting, reflecting extensive pull-out of TVBs (L, arrows).

We further experimentally studied the mechanical properties of the node, focusing on the roles of the discontinuous CVBs at the peripheral diaphragm (Fig. 3D–I) and the TVBs at the node culm (Fig. 1E–H). Although these specially oriented VBs do not make up a large proportion of the node, functioning as key minorities, they are expected to play important roles in maintaining the structural stability of bamboo. Three-point bending tests were carried out on the peripheral zone and central zone of the diaphragm (Fig. S17A and B). As shown in the fracture sections of the peripheral diaphragm (Fig. S17C and D), the discontinuous CVBs (indicated by yellow arrows and circles) surrounded by PCs exhibit pull-out and fracture behaviors. Their rough fracture surfaces (indicated by yellow arrows) further mean the slippage and pull-out of the constituent microfibers or more-refined twist-aligned nanofibers. These hierarchical fibrous CVBs are expected to guide crack propagation with a spatially tortuous path (Fig. S17B–E) [14,22,29–31]. However, for the central diaphragm with no directional fiber reinforcement, the crack appears relatively straight (Fig. S17B), suggesting low damage tolerance. As expected, the flexural stress and energy absorption of the CVB-dominated peripheral diaphragm are 60 MPa and 600 N mm, which are higher than those of the IVB-dominated central diaphragm (40 MPa and 400 N mm). Lateral wind load is one common load that bamboo bears; we further try to simulate it by radially compressing arched internode and node (containing little peripheral diaphragm) specimens to explore the discontinuous CVBs’ reinforcing effect (Fig. 5A). As shown in the fracture sections and reconstructed 3D crack of the node specimen (Fig. 5B)and C, and Movie S8), the discontinuous CVBs can still be pulled out and fractured, and can induce crack deflection (indicated by yellow arrows and circles). They show toughening behaviors like those of natural nacre [1,14,32–34]. In contrast, the arched internode specimen shows brittle fracture and poor mechanical properties. Its compressive load and energy absorption are 260 N and 200 N mm, which are much lower than those of the node specimen (800 N and 400 N mm) (Fig. 5A)and Fig. S18). Although it is difficult to achieve uniform specimen sizes between the arched internode and node (due to the presence of the peripheral diaphragm), the peripheral diaphragm accounts for a small proportion of the tested node specimen, and the specimen size effect can be ignored. In this case, these experimental results still highlight the mechanical roles of the discontinuous CVBs and show similarity with the numerical models (Fig. S15).

Uniaxial compression tests were carried out on the internode culm and node culm to study the influence of the TVBs on mechanical properties. As the load increased, the highly anisotropic internode culm showed gradual deformation until integral failure occurred (structural instability); AVBs fractured in the middle and PCs tore (Fig. 5D–F and Fig. S19A–E). For the node culm, the tightly interlocked structure (constructed by TVBs and AVBs) reduces the degree of anisotropy of the culm and can be considered as the reinforcement for AVBs in their weak direction. In this case, the presence of TVBs is expected to impede the serious damage of AVBs via ‘blocking effect’ and limit the damage to the position above TVBs (Fig. 5D, G and H, and Fig. S19F–J), enabling local failure or delayed failure of the node culm. This act of hindering damage propagation throughout the node culm can be manifested in practical mechanical tests: the appearance of quadratic peak load in the compressive stress-strain curves and large energy absorption of the node culm specimen (14.35 MJ m^−3^, higher than that of the internode culm (10.8 MJ m^−3^)) (Fig. 5D)and Fig. S20A). It is worth noting that the TVBs zone locates in the middle of the node culm specimen and occupies only a portion of the specimen volume. In this case, the applied compressive load can still fracture AVBs. Therefore, the compressive strength limits of internode and node specimens show consistency (∼60 MPa) (Fig. S20B). The measured strength values are similar or comparable to those of many bamboos previously reported (Table S3). To study the reinforcing effect of TVBs more visually, splitting tests were carried out on the internode culm and node culm (load direction parallel to axial direction and perpendicular to radial direction) (Fig. 5I). As shown in the multiscale fracture morphology and reconstructed 3D crack of the node culm (Fig. 5J)and L, Fig. S21A and B, and Movie S9), TVBs exhibit typical pull-out and fracture signals and can redirect crack propagation. The rough fracture surfaces of TVBs (Fig. S21A and B) further indicate the slippage and pull-out among the constituent microfibers or nanofibers. These multiscale motions of the fibrous TVBs partly reflect elegant nacre-like toughening behaviors that are activated via microplates’ and more-refined nanoparticles’ motions [1,14,34,35]. However, for the internode culm, a straight crack plane exists between AVBs and PCs (Fig. 5J)and K, and Fig. S21C and D) due to the anisotropic structure lacking transversal reinforcement. The splitting resistance of the node culm, therefore, is speculated to be higher than that of the internode culm. As expected, the energy absorption of the node culm and internode culm was measured as 280 N mm and 160 N mm, respectively (Fig. 5I)and Fig. S22). Similarly, the reinforcing effect of the TVBs can also be verified by three-point bending tests (Fig. S23). Frankly, the existing testing schemes are still very different from the real and complex bearing environments (gravity, sleet, wind, human intervention, etc.) of bamboo and its nodes. To more realistically understand the relationship between biological hierarchical fiber structures and properties, related work still needs to be done.

### Multiscale and multidirectional channels of the node culm

**Figure 6.**
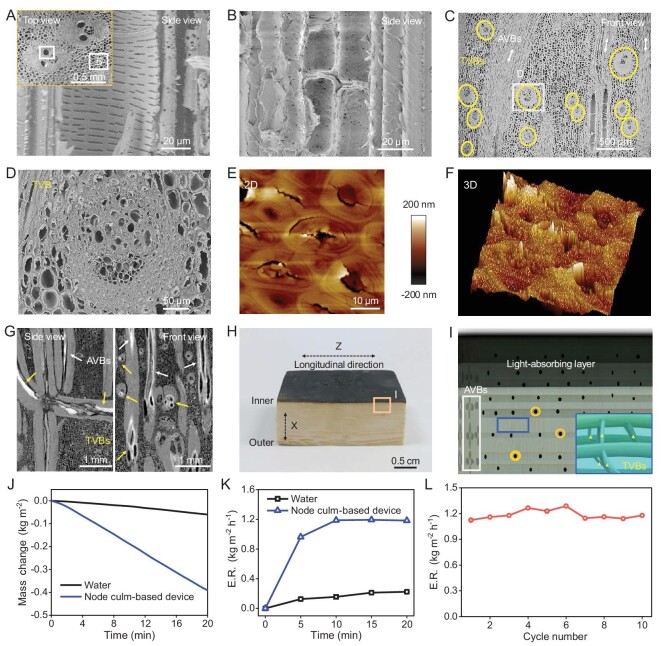
Multiscale and multidirectional channels of the node culm and application. (A and B) SEM images of multiscale channels including vessels, sieve tubes, conducting cells from one AVB (A) and pits from PCs (B). Inset (A) shows sampling sites. (C and D) SEM images of TVBs indicated by circles (C), showing abundant channels (D). (E and F) AFM images of the microfibers that make up one TVB show many micro-cracks and large geomorphologic fluctuation, reflecting the loose and porous characteristics of TVBs. (G) Slice projections of the node culm (after infiltrating copper nitrate solution and drying), showing that copper nitrate substances (bright zone) occupy the channels within the TVBs (yellow arrows) and AVBs (white arrows). (H) Digital image of a water evaporation device made from one node culm. The top layer is a carbonization zone (light-absorbing layer). (I) Schematic diagram of the water evaporation device, showing multiscale and multidirectional channels for transport. The white box shows AVBs and related channels, yellow circles show pits, and blue boxes and yellow triangles show TVBs and related channels. (J) Water mass change and (K) evaporation rate (ER) during device operation under 1 sun illumination. (L) Stability of water evaporation tests of the device (working 20 min and pausing 10 min as a cycle).

Multifunctional integration with minimal material consumption is a feature of biological materials. As mentioned above, the sophisticated arrangement of the VBs (such as TVBs) at the node is of vital importance to maintaining the structural stability of bamboo. Along with PCs, these mechanically strong fibrous VBs have directional channels (such as multiscale sieve tubes, vessels and pits) for liquid transport (Fig. 6A)and B, and Fig. S2). Note that many TVBs are at the key positions connecting lateral branch–culm–diaphragm, whose liquid and ion transport function is speculated to be prominent. As expected, SEM observations of TVBs show many micro-channels (Fig. 6C)and D, and Fig. S21B). AFM observations further reveal large in-plane geomorphologic fluctuation and some micro-cracks on the microfibers (Fig. 6E)and F), which is different from the geography of the microfibers from AVBs (Fig. S5A and C), hinting at the loose and channel-rich characteristics of TVBs. Here, with the rule that high atomic number materials are more capable of absorbing X-rays, we studied the multidirectional water passages of the node by tracking markers via the micro-CT technique. The dry rectangular node block was partly immersed in a copper nitrate (marker) aqueous solution, which can easily infiltrate into the channels under capillarity and low-pressure environments. After solvent evaporation, the inorganic salt remaining in the node can be detected by X-rays. The result shows that many channels from TVBs (yellow arrows) and AVBs (white arrows) are occupied by inorganic markers (bright areas) (Fig. 6G), indicating that multidirectional channels participate in liquid and ion transport. Here, due to the limited resolution of the micro-CT technique, the channels indicated by arrows come from VBs rather than tiny pits. It is worth noting that channels are not only related to liquid transport but also thermal conduction. The structure dominated by the AVBs and concomitant sieve tubes and vessels endows the node culm with anisotropic thermal conductivity: the axial thermal conductivity was tested to be 0.344 W m^−1^ K^−1^, while the radial thermal conductivity was 0.225 W m^−1^ K^−1^ (Fig. S24A). Compared to the case of wood, the degree of anisotropy of the node culm's thermal conductivity is low [36], implying the presence of transversal passageways (channel-rich TVBs). Interestingly, the radial thermal conductivity and the tangential thermal conductivity are similar (Fig. S24A), which further reflects that these transversal passageways are twisted, bifurcated and in-plane isotropic.

### Multifunctional integration of the node and potential application

Engineering applications can be inspired by biological materials, but engineering design sometimes cannot completely mimic them. The unidirectionally fibrous internode enjoys a high reputation for structural materials, while the node is always ignored or discarded. In fact, the node culm integrates many favorable properties including excellent structural stability (determined by interlocked reinforcement), sufficient water passages (supplied by multidirectional channels) and anisotropic thermal conductivity. With these structure-associated properties, eco-friendliness and sustainability, the node culm is expected to occupy its position in engineering practices, such as developing solar thermal water evaporation devices [37–39]. As proof of concept, a water evaporation device was designed using a modified node culm. The modification lay in the removal of the outer and inner sides of the node culm to expose multidirectional channels, and in the carbonation, which was used to prepare a light-absorbing layer (Fig. 6H). The hierarchical channel schematic diagram is shown in Fig. 6I. Axial channels (indicated by the white box) can not only transport water from both (left and right) sides but also function as heat insulators to promote heat utilization at the top light-absorbing layer. Transversal channels from TVBs (blue box and yellow triangle markers) and pits (yellow circles) can effectively transfer and gather water for evaporation. As shown in Fig. 6J–L and Fig. S24B–D, the water evaporation rate of the smoothly running device reaches 1.2 Kg m^−2^ h^−1^, which is comparable to that of many carbon-, polymer- and metal-based devices [40]. The device organically integrates modules that perform different functions. It is distinguished from not only synthetic devices relying on physical assembly and risking interfacial and structural disintegration [41,42] but also the anisotropic wood- and sugarcane-based devices risking directional erosion during long-term waterborne service [36,42,43]. The excellent splitting resistance of the node culm partly reflects the device's structural advantage in dealing with directional erosion (Fig. 5I). The experimental splitting loading on the wet internode culm and node culm (specimens were soaked in flowing water for 10 days) further confirmed this advantage. As shown in Fig. S25, the peak load and the energy absorption of the wet node culm are 280 N and 485 N mm, which are higher than those of the wet internode culm (180 N and 240 N mm). Overall, the device organically accumulates the merits of mechanical reinforcement, liquid transport and thermal conduction, and demonstrates the node's multifunctional integration. It can be predicted that the exploration of more sophisticated biological structures will help promote progress in engineering.

### Extended perspective and discussion

For the promising bamboo node, instead of applying static and short-time loads for testing, we tentatively applied a continuous constant load (300 N, elastic range for node) to explore its mechanical response. The result shows that the node deforms 0.076% during the 4400 seconds of applying a 300 N constant load (Fig. S26A and B). We changed the deformation-time curve to the logarithmic form and found that the curve tends to be a straight line (Fig. S26C). Fitting the curve, we further found that the slope (power exponent) is ∼0.2 (Fig. S26D). These results can partly show that the creep behavior of the bamboo node follows a power-law response. It is worth mentioning that the deformation-time response and the power exponent of the bamboo node are like the cases of biological cells. According to previous reports, regardless of cell types or states, the mechanical behaviors of cells follow a power-law relationship, and the power exponent is mostly between 0.1 and 0.5, possibly due to the self-similar hierarchical structures [44–48]. The power exponent of the bamboo node also falls in this range, which can be explained by the proposed self-similar hierarchical model [44], as the bamboo node can be treated as a four-level self-similar hierarchical structure: the combination of the three-level model for the cell and a higher-level hierarchy for the fiber and matrix or tissue. It can be envisioned that the mechanical behaviors at the cellular scale can transfer to the macroscopic scale (cell assemblies), which will facilitate the development of biology, biomechanics and biomimetic materials [47].

Another consideration is the moisture effect on the mechanical properties of biological materials, which has attracted wide attention [49–52]. For the bamboo that consists of cellulose, semi-cellulose, lignin and channels, moisture can easily infiltrate into the multiscale fiber interfaces and channels. It can influence the destruction and reconstruction of interfacial hydrogen bonds and facilitate hierarchical vascular bundles’ and parenchyma cells’ motions (such as slippage, pull-out, twist, bridging, buckling and cracking). In this work, the mechanical properties of the dry (10.65 wt.% moisture content) and wet (51.11 wt.% moisture content) specimens were studied under splitting loading (Fig. 5I)and Figs S22 and S25). Whether it is node culm or internode culm, the dry materials exhibit brittle fracture with high peak load (strength and modulus) and low energy absorption (Fig. 5I)and Fig. S22). The wet materials exhibit ductile failure with low peak load, large deformation and high energy absorption (Fig. S25). It can be found that with the increase of moisture content, the materials gradually change from brittle failure to ductile failure. Systematic studies of the moisture effect on the mechanical properties of biological materials are ongoing.

## CONCLUSION

In this work, we put the sophisticated arranged VBs of the bamboo node into several categories (such as AVB, TVB, CVB, RVB and IVB), and extracted and verified three kinds of fiber reinforcement schemes, including interlocked structure, spatial scaffolding structure and isotropic intertwined structure. These fiber-reinforced structures are hierarchically assembled by cellulose molecules, nanocrystals, nanofibers, microfibers and VBs. Multiscale toughening mechanisms (such as slippage, pull-out and rotation of nanofibers, microfibers and VBs) will synergistically play roles in the node's mechanical robustness. In addition, we experimentally found that the multidirectional channels within the node can transport liquid. Further combining the node's functions of mechanical reinforcement, multidirectional liquid transport and anisotropic thermal conductivity, we designed a structurally stable and high-performance solar thermal water evaporation device and demonstrated the node's capabilities. Our findings could provide insights that will inform engineering structural design, biomass utilization and sustainable development. Structural materials (such as lightweight connecting materials and fatigue-resistant energy-absorbing materials), water treatment and optothermal management devices, and ionic transport devices (such as electrodes), can all be envisioned. Learning from nature is an eternal theme, and researchers should constantly strive to explore it.

## METHODS

### Materials

All the chemicals are purchased from Sinopharm Chemical Reagent Group and used as received. Moso bamboos (*P. edulis*) are mainly collected from Hefei Botanical Garden. The age of bamboos is ∼5 years old, and the collection season is summer. The most used specimens (including internode culm and node culm) were taken from the middle (both height direction and radial direction) of bamboos.

### Mechanical tests

All the mechanical tests are carried out on Instron 5565 A equipment using 500 N and 5000 N load cells. For the splitting tests, the specimen size is 10 mm (Z, axial direction) × 8.5 mm (X, radial direction) × 8 mm (Y, tangential direction). The loading speed is 0.5 mm min^−1^, and the load direction is parallel to the axial direction and perpendicular to the radial direction. For the compression tests of the culm-related semi-circular arched specimens, the specimen size can be featured with 20–22 mm (height, circular radius), ∼12 mm (width, axial direction), 7–9 mm (culm thickness) and 14–16 mm (total thickness). The loading speed is 0.01 mm s^−1^. For the three-point bending tests of the diaphragm, the specimens are carefully cut to 4 mm thickness (axial direction) and 3.5 mm width. The span is 10 mm, and the loading speed is 0.1 mm min^−1^. For the three-point bending tests of the culm, the specimens are carefully cut to 3 mm thickness (axial direction) × 5.5 mm width (radial direction). The span is 5 mm, and the loading speed is 0.1 mm min^−1^. For the uniaxial compression tests of the culm, the specimen size is 6 mm (axial direction) × 5 mm (radial direction) × 5 mm (tangential direction). The loading speed is 1 mm min^−1^, and the load direction is parallel to the axial direction. Micro-indentation (Vickers hardness) tests are carried out using a microhardness tester (EMCOTEST Durascan) with an applied load of 0.2 kgN for 5 s. All specimens were stored at a relative humidity of 40% at 25°C for 24 h before tests. All mechanical experiments were carried out at room temperature (23–27°C) and humidity (40%–50%).

### Sample characterizations

Silicon carbide sandpaper of different meshes (1000, 3000, 5000, 7000) are gradually used to polish the dissected bamboo specimens. Optical observations are carried out with a microscope (OLYMPUS BX53M). SEM images are acquired by instrument (Hitachi, SU8220) at an acceleration voltage of 3 kV. All specimens are coated with platinum (2 nm thickness) before observation. Micro-CT experiments are carried out with an X-ray microscope (Zeiss, Xradia 520 Versa), and related images and movies are acquired by Dragonfly software. AFM images are acquired by the Bruker Dimension Icon using Peak Force Tapping Mode (morphology test mode, using ScanAsyst-Air AFM tip) and Peak Force QNM mode (mechanical modulus test mode, using REFSPA-525 AFM tip). The thermal conductivity of specimens (∼10 mm × 10 mm × 1 mm) is measured using laser thermal conductivity apparatus (LFA467, NETZSCH). Infrared images of specimens are taken by a thermal infrared camera (ICI-7320).

## Supplementary Material

nwac195_Supplemental_Files
