# Supplementary material for: Mechanically robust bamboo node and its hierarchically fibrous structural design
Source: Natl Sci Rev. 2022 Sep 22;10(2):nwac195. doi: 10.1093/nsr/nwac195 (PMC9935994; doi:10.1093/nsr/nwac195)
Supplement: nwac195_Supplemental_Files [file nwac195_supplemental_files.zip › Supplementary data.pdf]

Supplementary Information for

Mechanically robust bamboo node and its hierarchically fibrous structural design

Si-Ming Chen, Si-Chao Zhang, Huai-Ling Gao, Quan Wang, LiChuan Zhou, Hao-Yu Zhao, Xin-Yu Li, Ming Gong, Xiao-Feng Pan, Chen Cui, Ze-Yu Wang, YongLiang Zhang, HengAn Wu, Shu-Hong Yu

Corresponding authors: Huai-Ling Gao and Shu-Hong Yu.
Email: ghuailing@ustc.edu.cn, shyu@ustc.edu.cn.

This file includes:
Supplementary text
Figures S1 to S26
Tables S1 to S5
Legends for Movies S1 to S9
References

Other supplementary materials for this manuscript include the following:
Movies S1 to S9

Supplementary Information Text

Experimental Section

Lignin removal for examining VB morphologies

3.0 g of sodium hydroxide and 1.5 g of sodium sulfite are dissolved in 30 mL of water, then 0.17 g of bamboo chips are put into the fore-mentioned solution and treated at 80 ℃ for 12 hours. After that, the softened bamboo chips are taken out and can be highlighted the fibrous VBs using tweezers.

Detection of multidirectional water passages

The outer surface (radial direction) of a dry node block (~6 mm × 6 mm × 6 mm) is immersed in copper nitrate solution (0.5 mol L^{-1}) for 20 minutes under a low-pressure condition, after which the block is taken out and surface residual solution is wiped. After drying, the block is subjected to a micro-CT experiment to study the distribution of copper nitrate crystals in the channels.

Solar steam generation experiments

A xenon lamp (PLS-SXE300D) and a light intensity meter (LP100) are used. The designed node culm-based device (2.5 cm (axial direction) × 2 cm (tangential direction) × 1 cm (radial direction)) is placed in a glass dish filled with water, and the outer surface (radial direction) is in contact with water. Only the carbonized surface (inner surface) of the device is exposed to light and another water surface is covered by foam. Continuous weight loss during steam generation under 1 sun is monitored using an electronic balance (AL204, METTLER TOLEDO).

Supplementary mechanical simulations

The Finite Element Method (FEM) is adopted to simulate the deformation behavior and reinforcing mechanisms of several fibrous VBs of bamboo under compression loads [1,2]. To simplify the complex model and improve convergence, the plane stress model is adopted. We use the 8-node biquadratic plane stress quadrilateral solid elements (CPS8) and consider the nonlinear effects of large

displacement. All simulations are completed by ABAQUS. Two sets of numerical models were constructed to investigate the reinforcing mechanisms of several fibrous VBs under longitudinal (axial) and in-plane compression load conditions.

In the first group, we constructed two models (Diagram 1) to qualitatively investigate the deformation of a single longitudinal fiber (Model#1) and the interaction between a single longitudinal fiber and a single horizontal fiber (Model#2), respectively. As a representative, the 3D model (at the left of Diagram 1) for fiber arrangement can be simplified to the 2D model to improve computational efficiency. The geometry and materials parameters of models were partly referred to experiments and listed in Table S4. In the compression process, the displacement of the middle point at the bottom edge of the longitudinal fiber, and the bottom and left edges of the horizontal fiber, were restrained in the Y and X-directions. The compression load was implemented by the displacement along the negative Y direction at the middle point of the top edge of the longitudinal fiber. Note that the VBs interlocked structure of node culm was confirmed by experiments, we here only extracted the basic fibrous units to qualitatively study the reinforcement caused by TVBs.

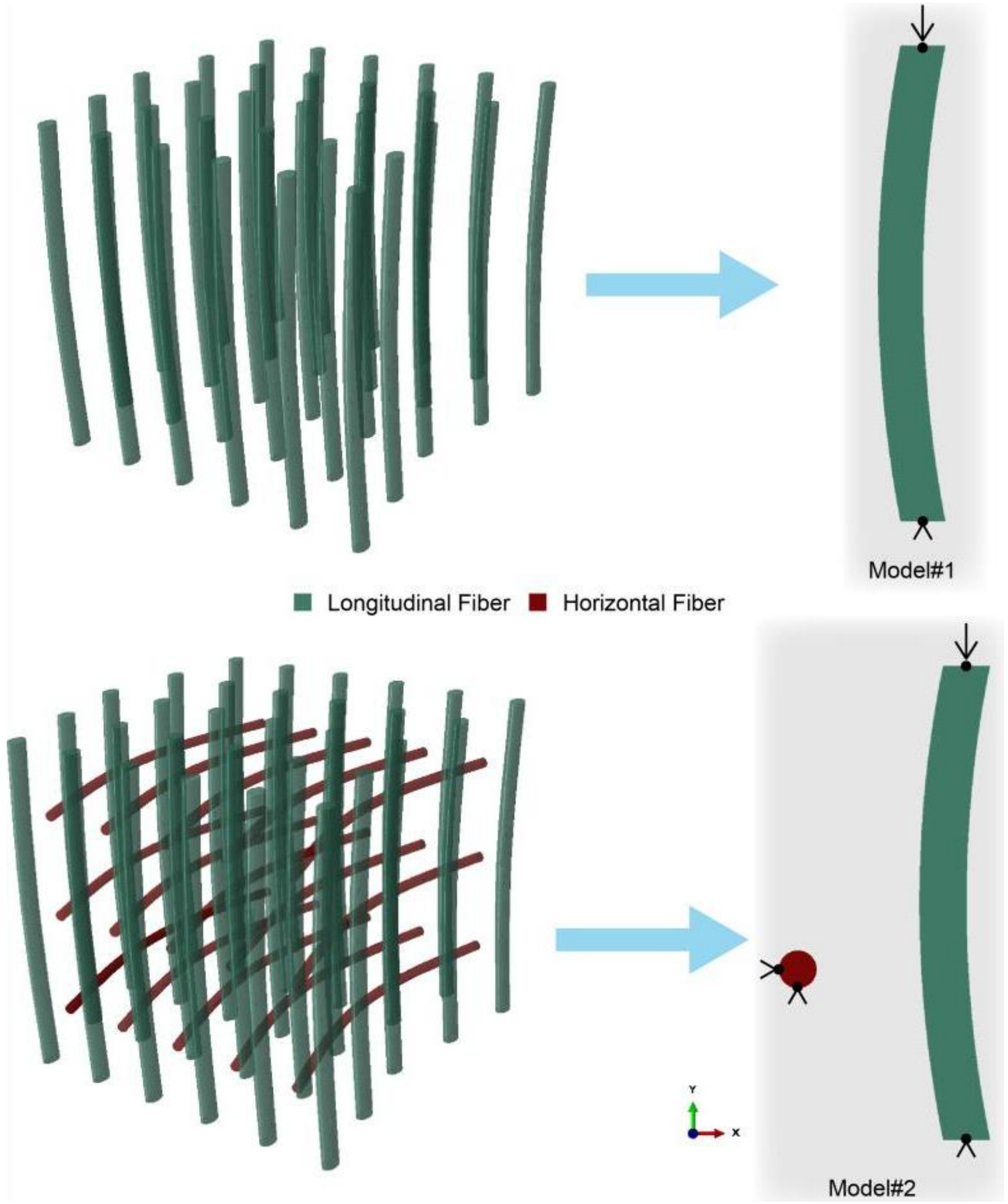

Diagram 1.

According to the actual structure of fibrous vascular bundles at the bamboo node, three idealized arrangements of fibers are proposed. As a representative, we constructed three models (Diagram 2) to qualitatively investigate the deformation and reinforcing mechanisms of several fibrous VBs at the diaphragm under in-plane compression load conditions. Only a quarter of the model was considered due to the symmetry of the problem. In Model#1, fibers were extended along the radius near the inner culm, while along the circumferential direction in Model#2. Compared with Model#1, fibers were radially extended to the center of the section in Model#3. The models kept the same fiber mass. The geometry and materials parameters of models were partly referred to experiments and listed in Table S5. In the compression process, the displacements at the left edges of the models were restrained in the X direction, and at the bottom edges were restrained in the Y direction. The models were compressed by a rigid plate along the negative Y direction from the top. For Model#1 and Model#2, the possible mechanical effect caused by the circumferential fibers can be explored under other equal conditions. For Model#1 and Model#3, we aim to explore the difference in mechanical reinforcement of these radially extended fibers either directly passing through the center, or only maintaining in the periphery combined with the central isotropic soft diaphragm.

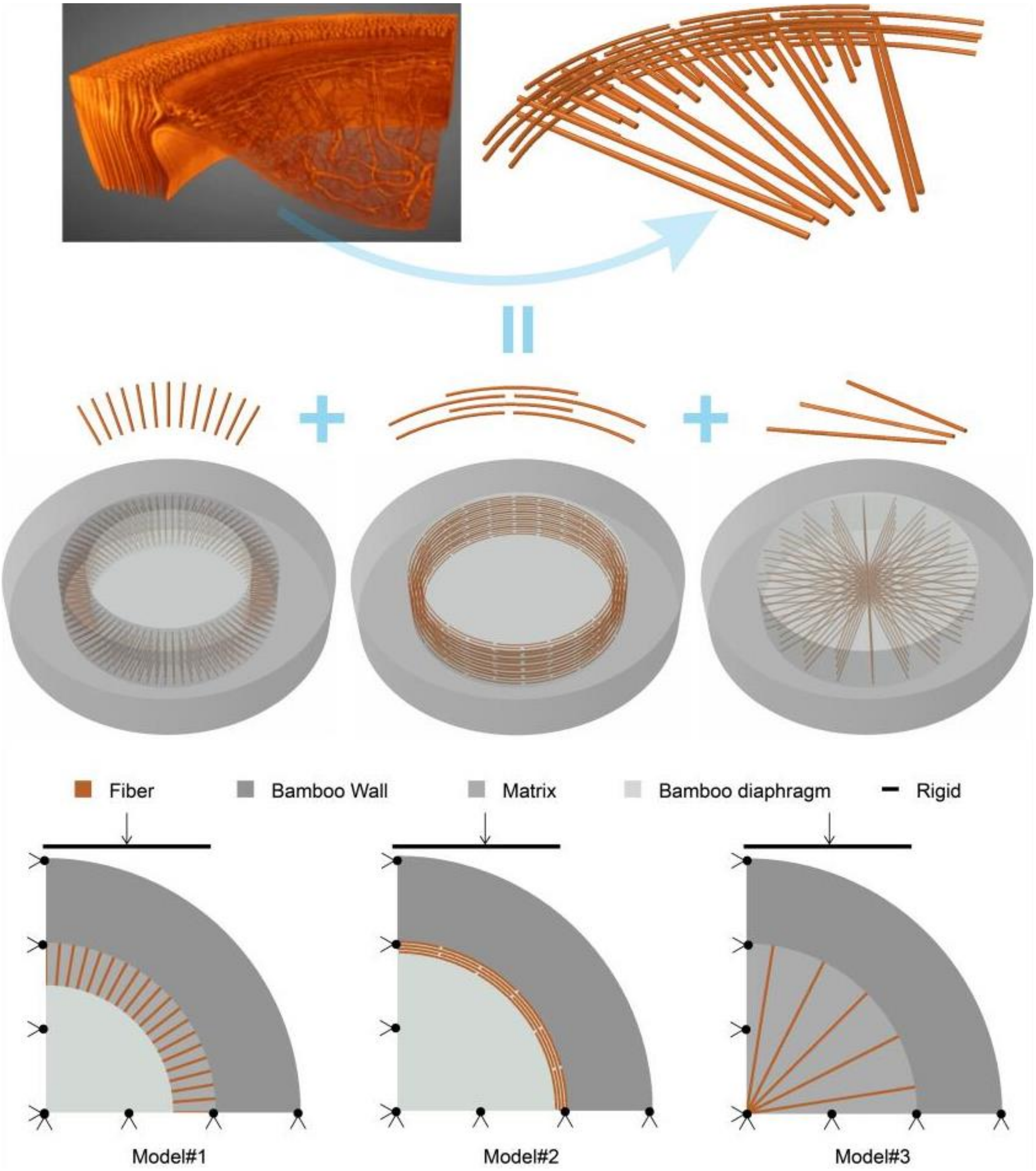
Fiber
Bamboo Wall
Matrix
Bamboo diaphragm
Rigid
Model#1
Model#2
Model#3

Diagram 2.

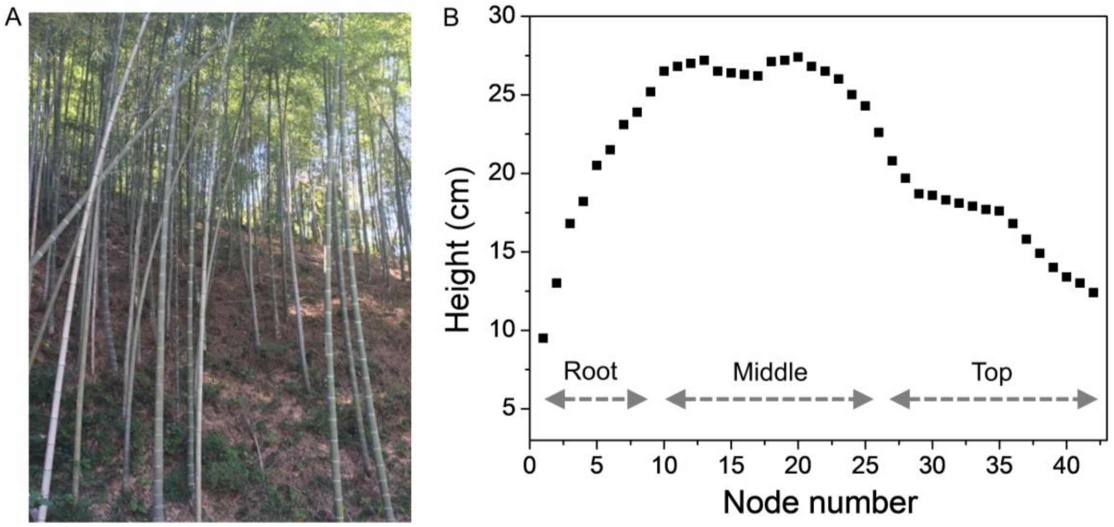

Fig. S1.
(**A**) Digital image of bamboo forest. (**B**) Height-node data plot of one bamboo. The root, middle and top regions of one bamboo can be preliminarily divided.

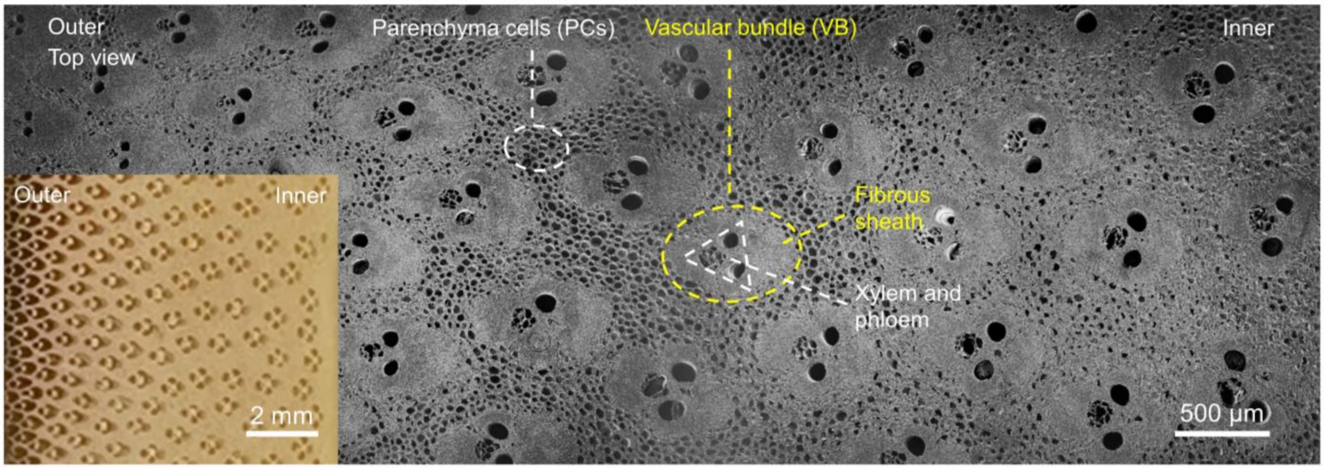

Fig. S2.
Structural characterization of the bamboo culm. SEM and OM (inset) images of culm, showing gradient distribution of VBs surrounded by PCs. VBs are sparse on the inner culm and dense on the outside, they mainly consist of xylem, phloem, and fibrous sheath.

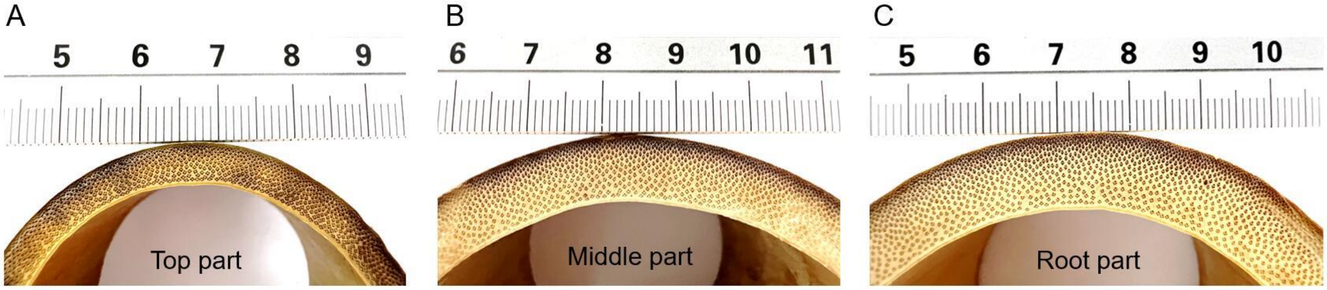

Fig. S3.
Digital images of culms from different parts (**A**-**C**), showing gradient distribution of VBs.

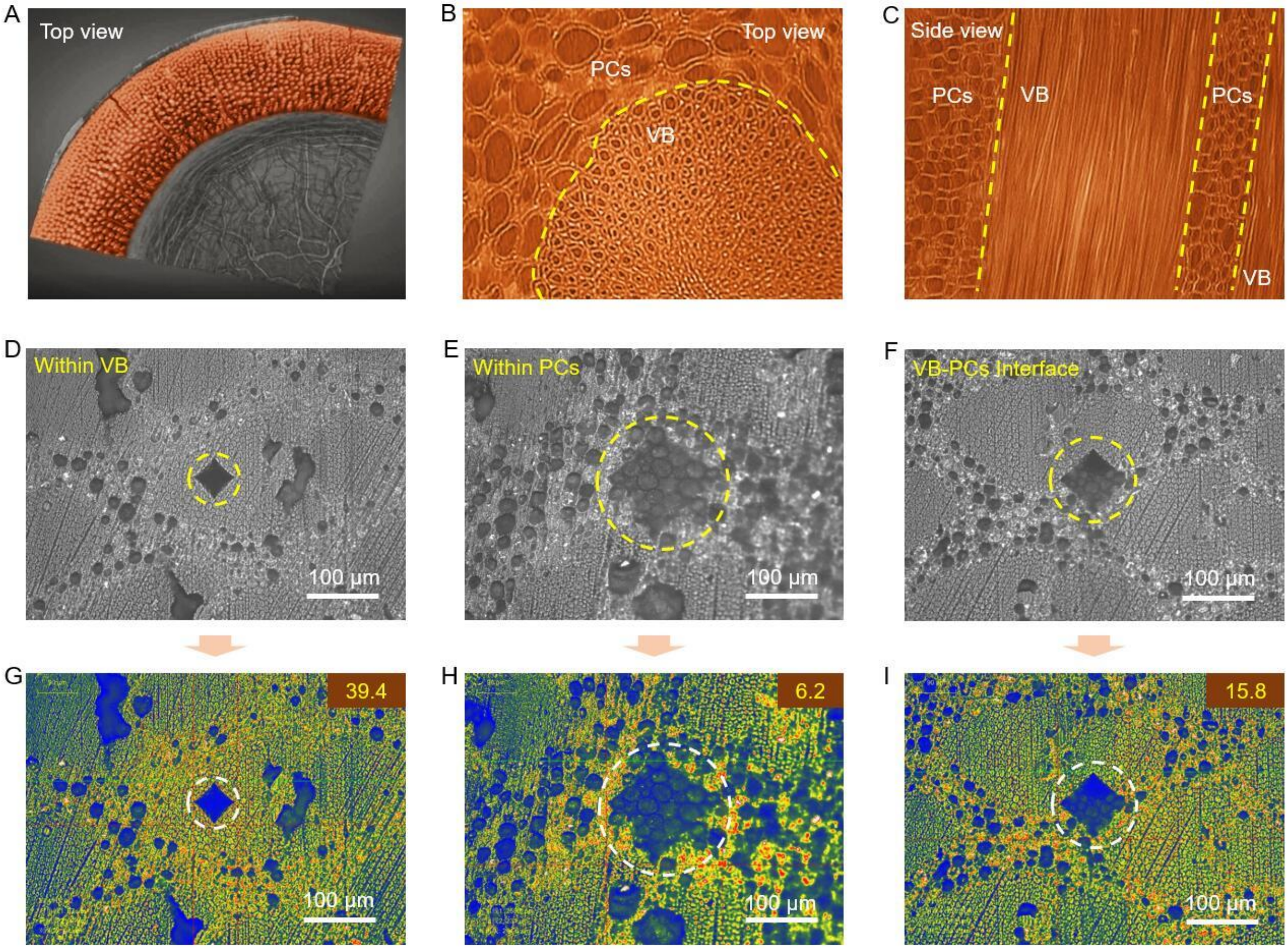

Fig. S4.
(**A**) Reconstructed 3D configuration of one node culm, showing gradient distribution of VBs. (**B-C**) Snapshots of the reconstructed 3D configuration of one VB and surrounding PCs. OM images and micro-indentation tests of culm including VBs (**D**,**G**), PCs (**E**,**H**), and their interface (**F**,**I**). Circle markers indicate indentation areas. Based on indentation sizes and related hardness values, it can be concluded that VBs are stronger than surrounding PCs, reflecting that bamboo is a typical fiber-reinforced composite.

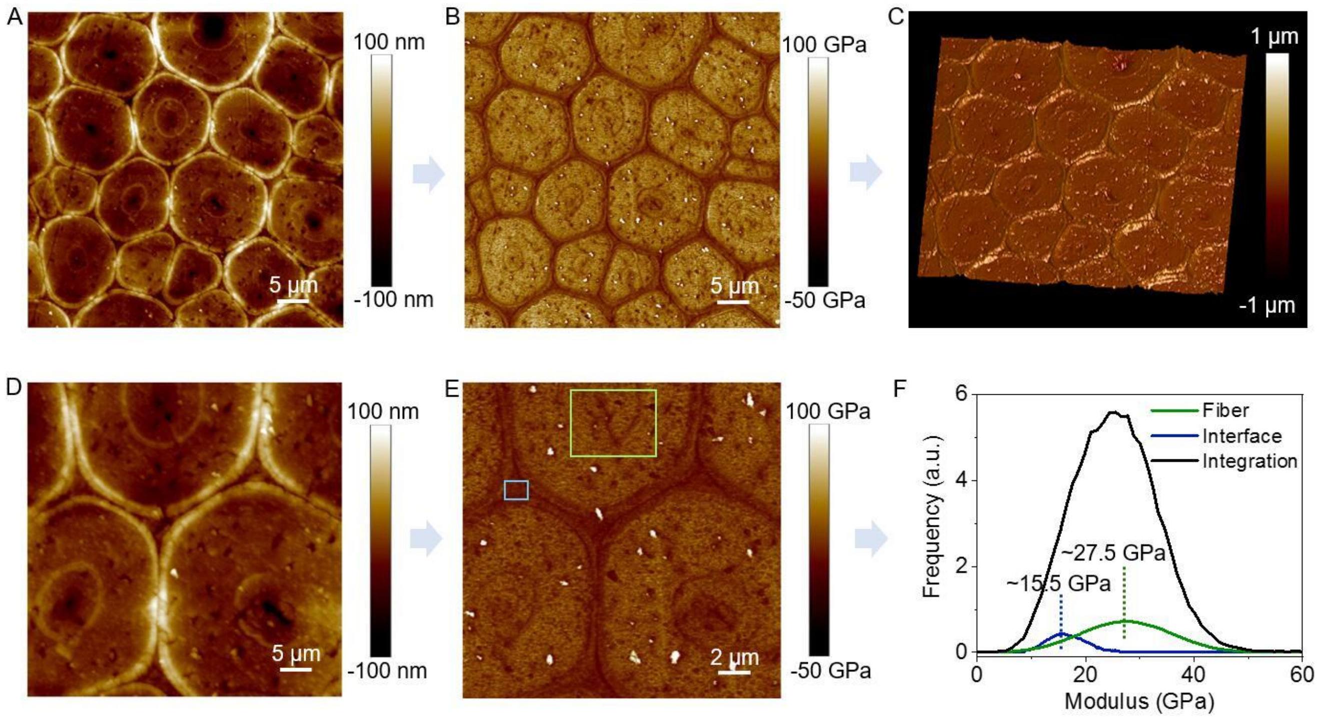

Fig. S5.
AFM images (**A**,**C**-**D**) and modulus mappings (**B**,**E**) of microfibers and interfaces that make up one VB. Small in-plane geomorphologic fluctuation means that these microfibers are dense without obvious pore (**C**). It can be seen that microfibers are stronger than their interfaces. (**F**) Statistical distribution of the moduli based on modulus mapping (**E**), two peaks can be identified for microfibers and interfaces, and corresponding moduli are ~27.5 GPa and ~15.5 GPa. The integration curve comes from the overall image (**E**) that removes light spots (impurities), the fiber curve comes from the green box (**E**), and the interface curve comes from the small blue box (**E**).

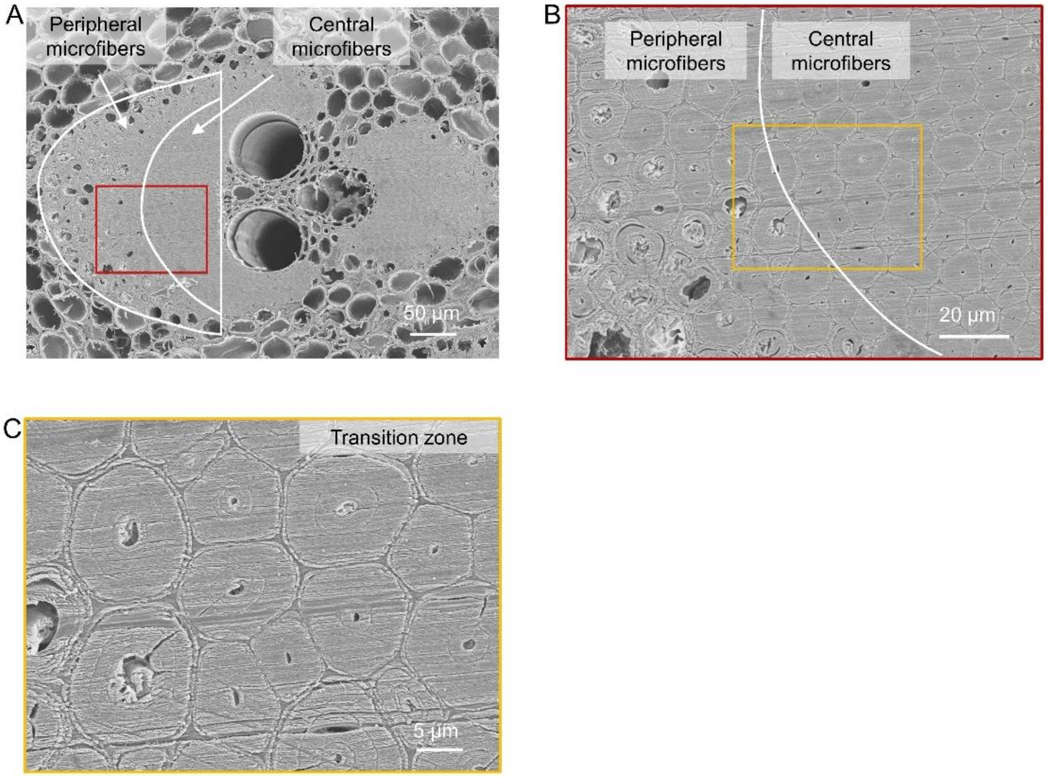

Fig. S6.
Structural characterization of the AVBs at the internal node culm. (**A-C**) SEM images of one AVB. The peripheral (outer) microfibers within the AVB display many micropores and sublayers, and central (inner) microfibers appear dense.

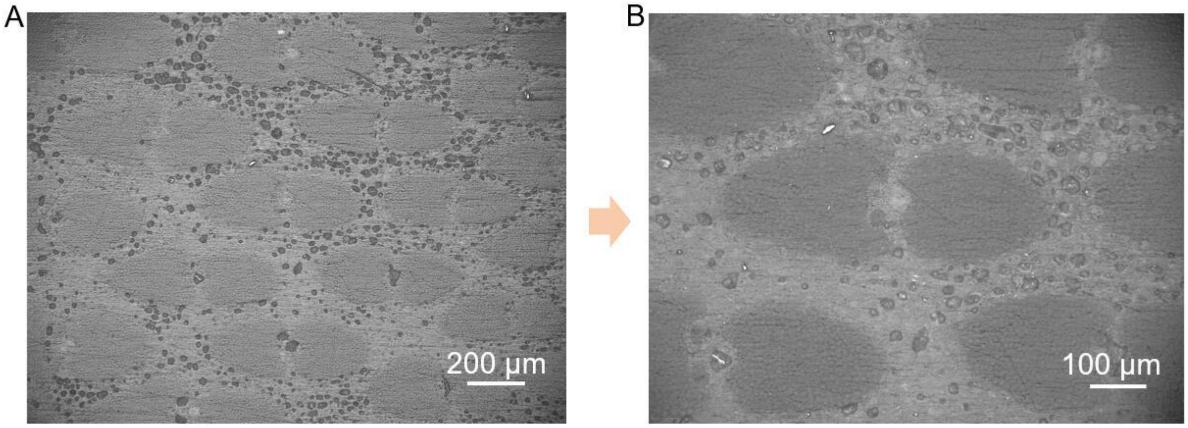

Fig. S7.
Structural characterization of the AVBs at the external node culm. (**A**-**B**) OM images of these AVBs, showing denseness.

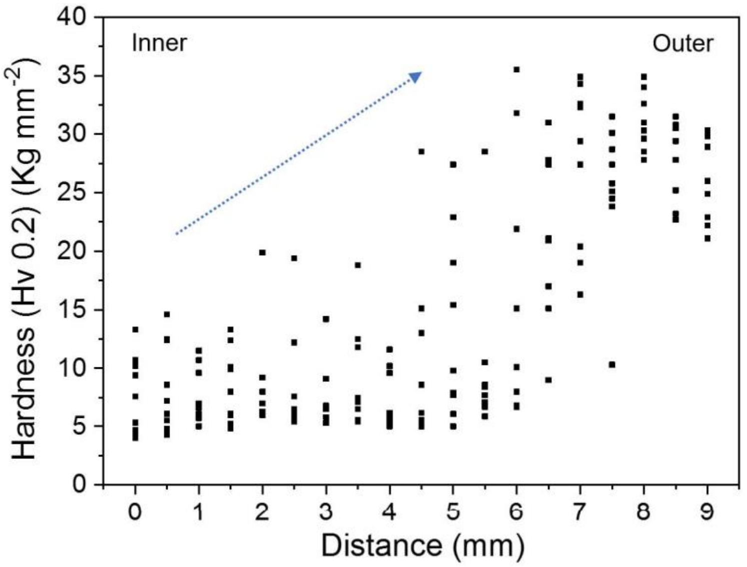

Fig. S8.
Hardness data plot of bamboo node culm in the radial direction (from inner to outer). The outer hardness is significantly higher than the inner hardness.

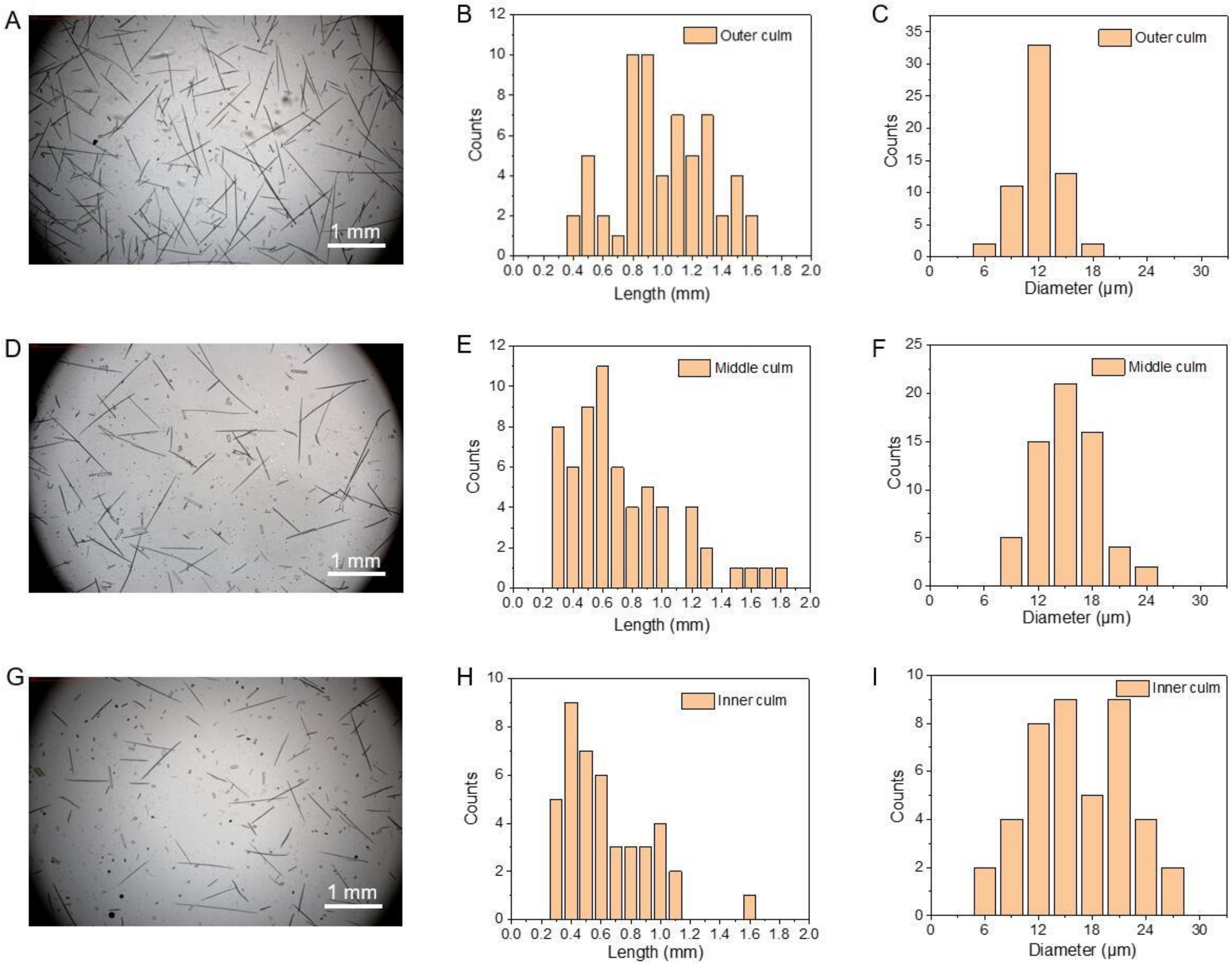

Fig. S9.
Optical image of the microfibers from position-dependent (outer-middle-inner) culms and microfiber length and diameter statistics.

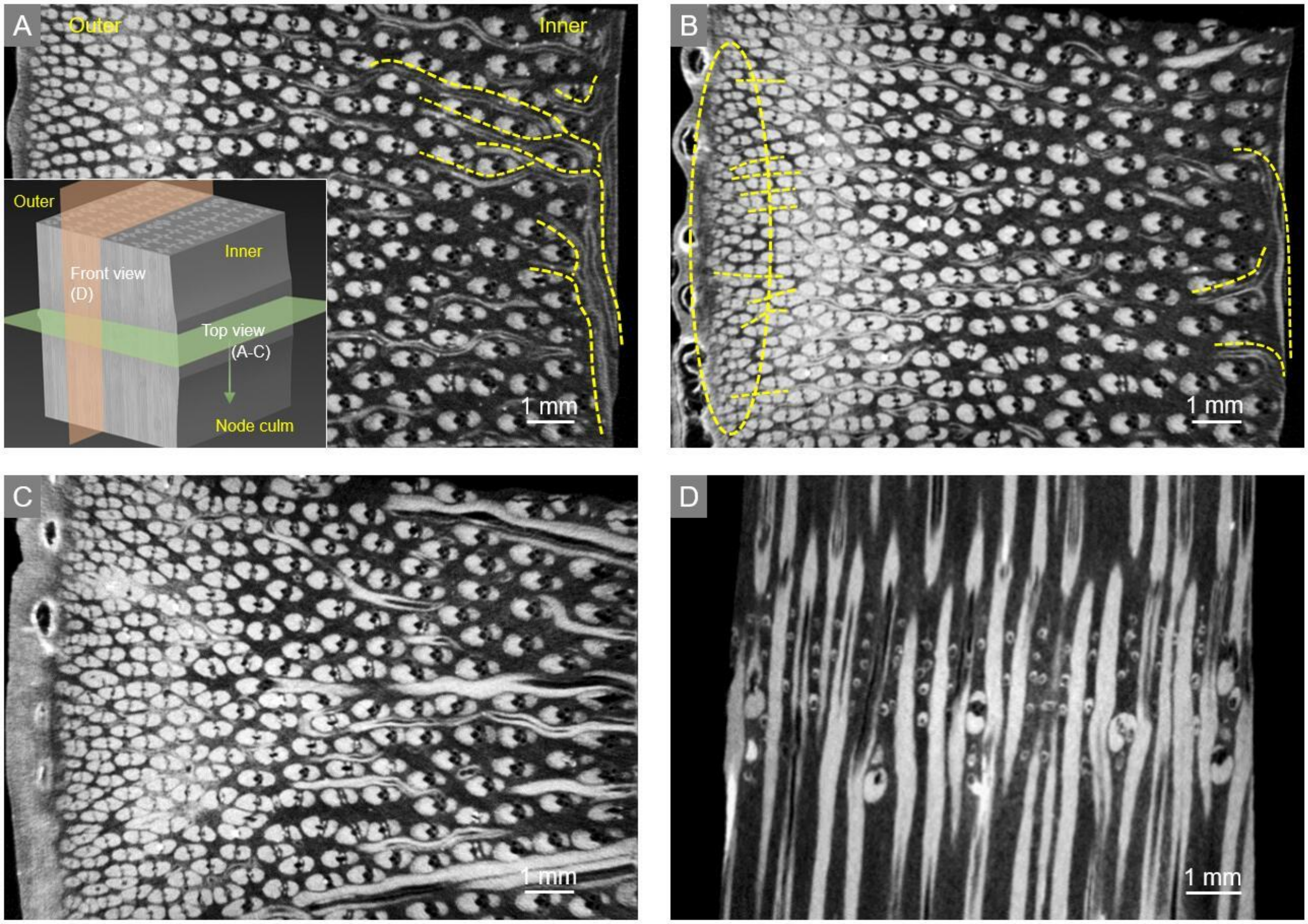

Fig. S10.
Diversity of TVBs. (**A**-**C**) Slice projections of node culm from top to bottom. Inset (**A**) is a schematic of slicing directions. Many TVBs are twisted and interlaced with AVBs. Some thin and high-positioned TVBs inward bifurcate and link the diaphragm resulting in circumferential arrangement (indicated by lines (**A**, **B right**)). These TVBs outward connect to the outside (indicated by a circle and some lines (**B left**)). The discontinuous presence of these TVBs partly reflects their spatial twisting. Some large, thick and low-positioned TVBs come from AVBs (**C**). (**D**) Slice projection of node culm, further showing the diversity of TVBs in terms of their size and position. AVBs are also partially distorted by intertwining and squeezing with TVBs (**D**).

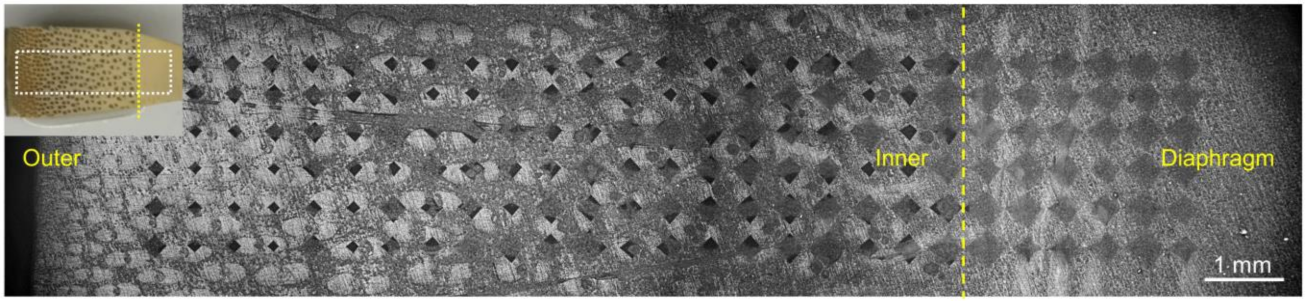

Fig. S11.
Micro-indentation marking array on the node including culm and diaphragm (top view). The difference in indentation sizes shows that the node culm is much stiffer than the diaphragm. Inset shows real specimen and indentation position (indicated by white box).

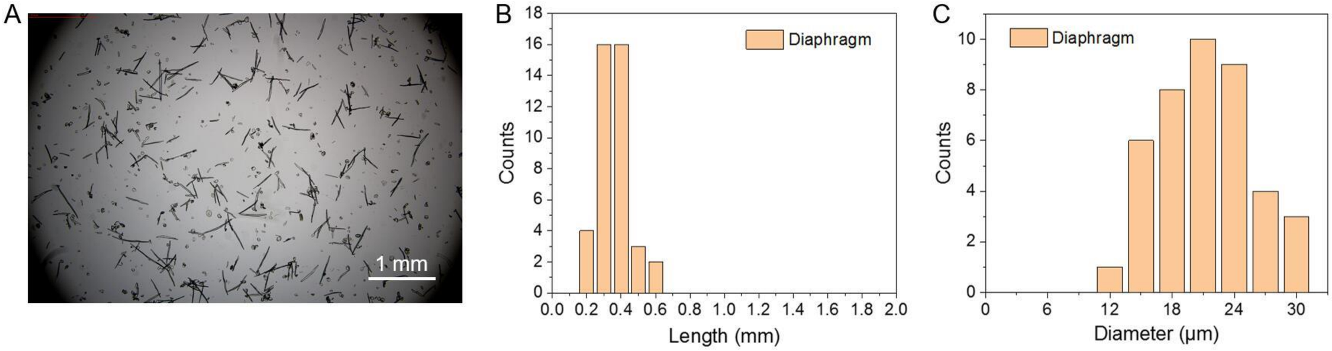

Fig. S12.
Optical image of the microfibers from diaphragm and microfiber length and diameter statistics.

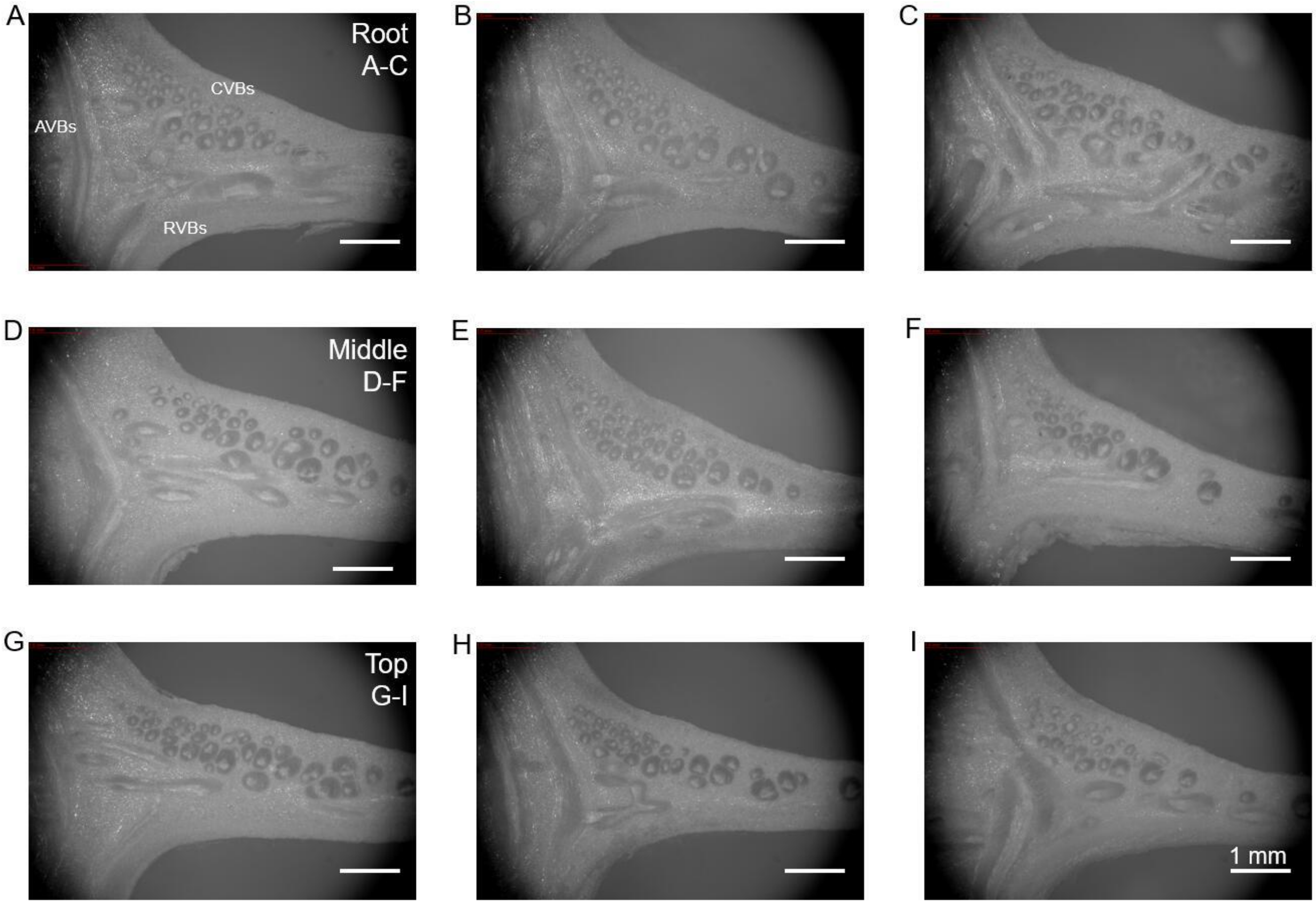

Fig. S13.
Optical images of the inner culm-diaphragm bridge zones from different height-located nodes. There are no obvious differences.

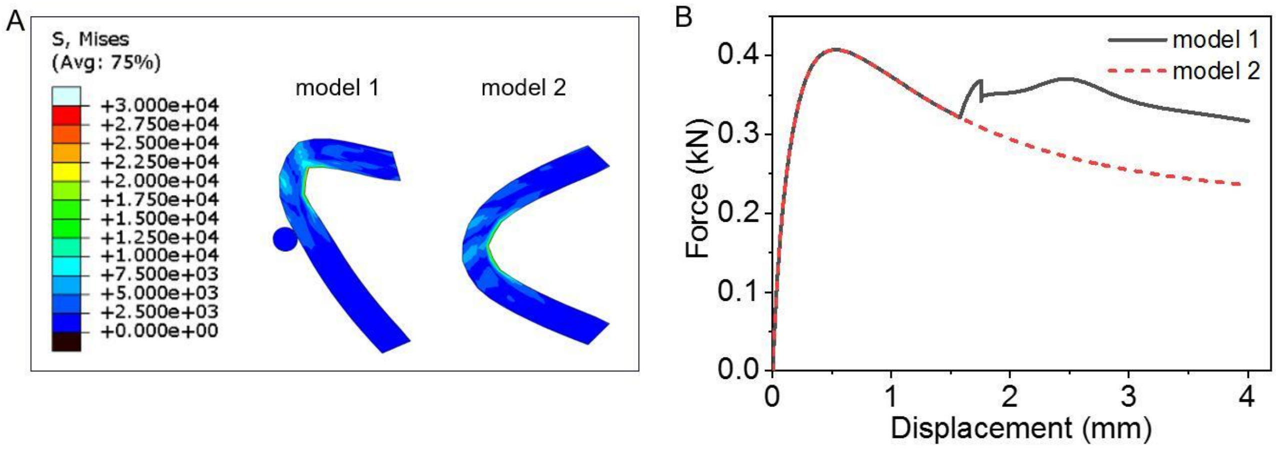

Fig. S14.
Mechanical simulations of node and internode culms. (**A**) Qualitatively simulated final states of node (model 1, the combination of the axial and transversal fibers) and internode (model 2, axial fiber) culms under compression load conditions. (**B**) Qualitatively simulated uniaxial compression stress-strain curves of these two models, demonstrating the mechanical advantage of the presence of transversal fiber. The end of the compression stress-strain curves mainly considers that the overall compression displacement was controlled to be equal.

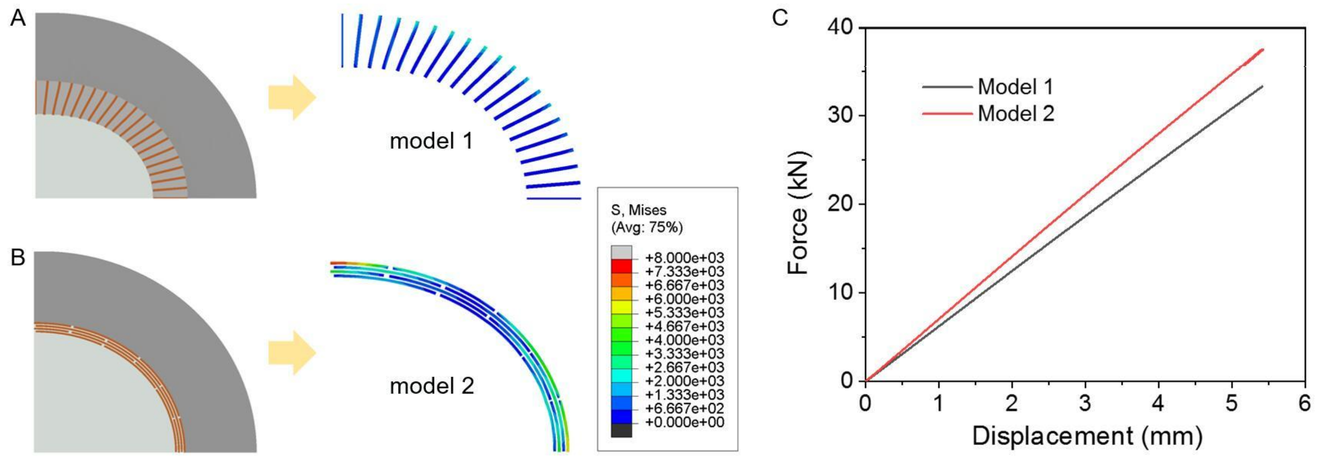

Fig. S15.
Qualitative simulations of nodes under in-plane compression load conditions. (**A**-**B**) Simulated final states of bamboo nodes. (**C**) Simulated compression stress-strain curves of bamboo nodes, demonstrating the circumferential fiber's mechanical advantage (increasing stiffness appropriately). The end of the compression stress-strain curves mainly considers that the overall compression displacement is controlled to be equal.

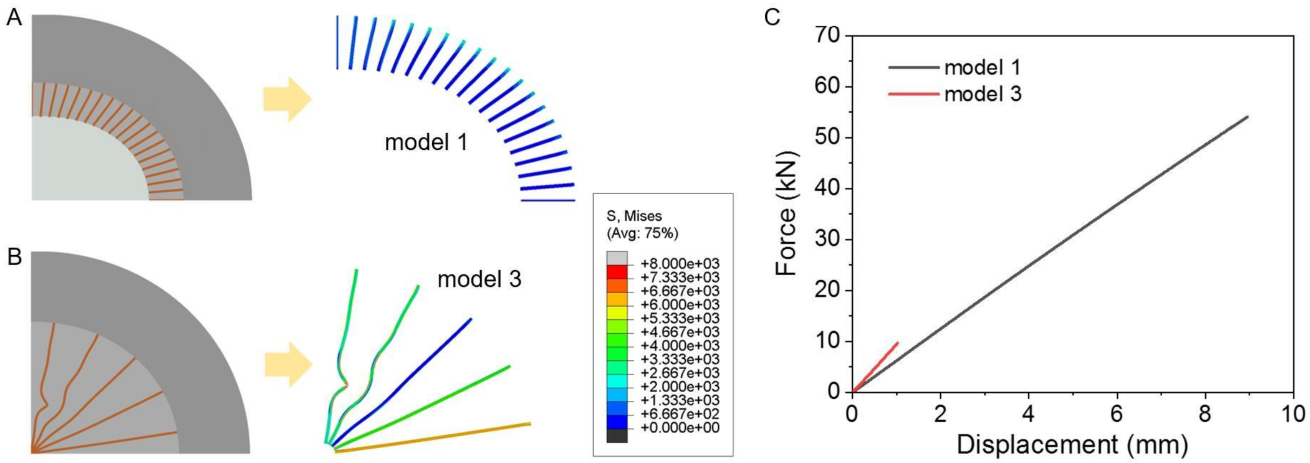

Fig. S16.
Qualitative simulations of nodes under in-plane compression load conditions. (**A**-**B**) Simulated final states of nodes. (**C**) Simulated compression stress-strain curves of bamboo nodes, demonstrating that the combination of locally radially penetrated fibers and central isotropic soft zone endows the mechanical advantages of avoiding premature failure and maintaining stability. The end of compression stress-strain curves mainly considers that the stress of the highlighted fibers in the two models is consistent.

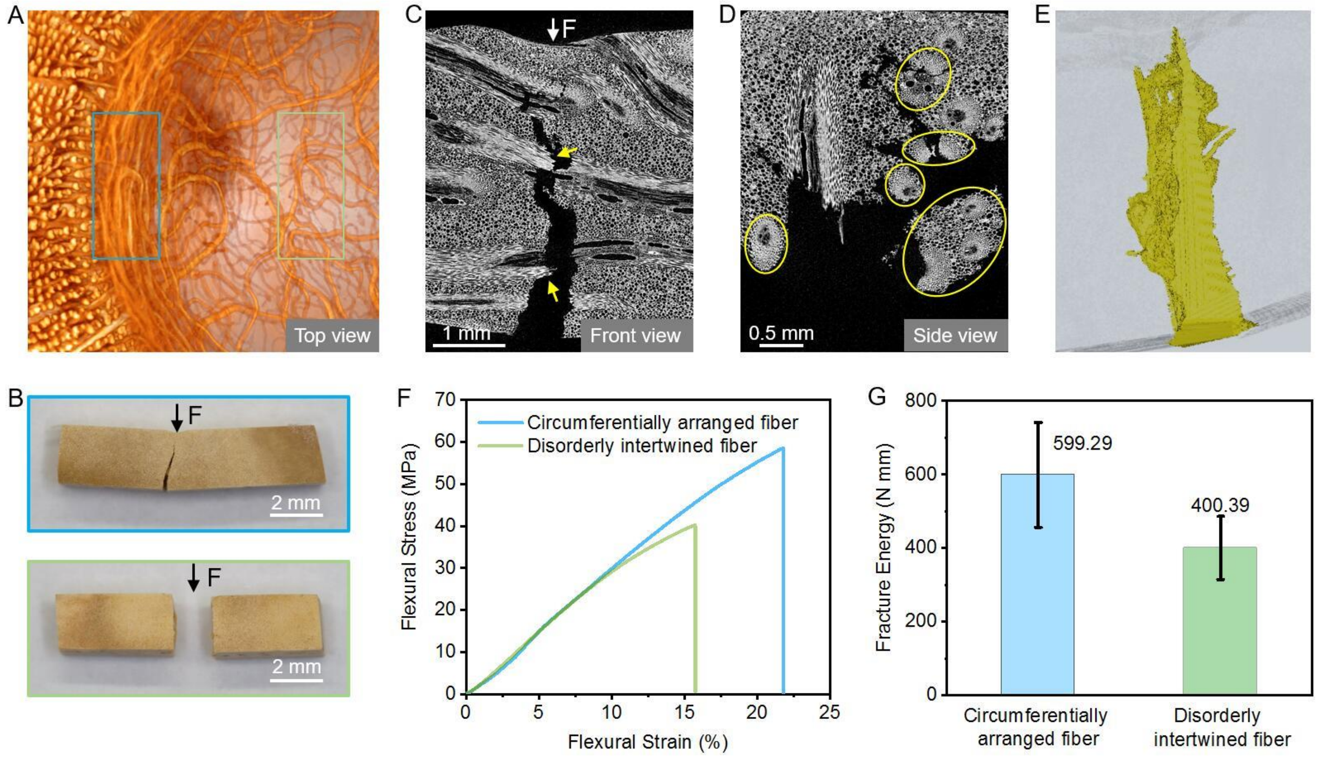

Fig. S17.
Three-point bending tests of the peripheral and central diaphragm. (**A**) The sampling sites for tests, blue and green boxes represent the periphery and center. (**B**) Real specimens of the peripheral (top) and central (bottom) regions after tests. (**C**-**D**) Slice projections of the peripheral region after the test. Circles (**D**) indicate cross-sections of VBs. Yellow arrows and circles (**C**-**D**) show the pull-out and fracture of the CVBs for toughening. (**E**) Reconstructed 3D crack showing complicated and twisted state. (**F**-**G**) Stress-strain curves and fracture energies of the peripheral and central diaphragm.

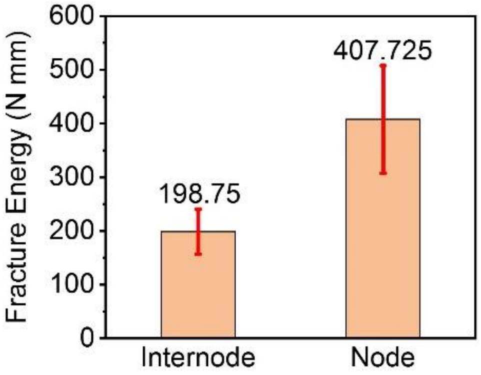

Fig. S18.
Fracture energies of internode and node culm-related arched specimens after compression tests.

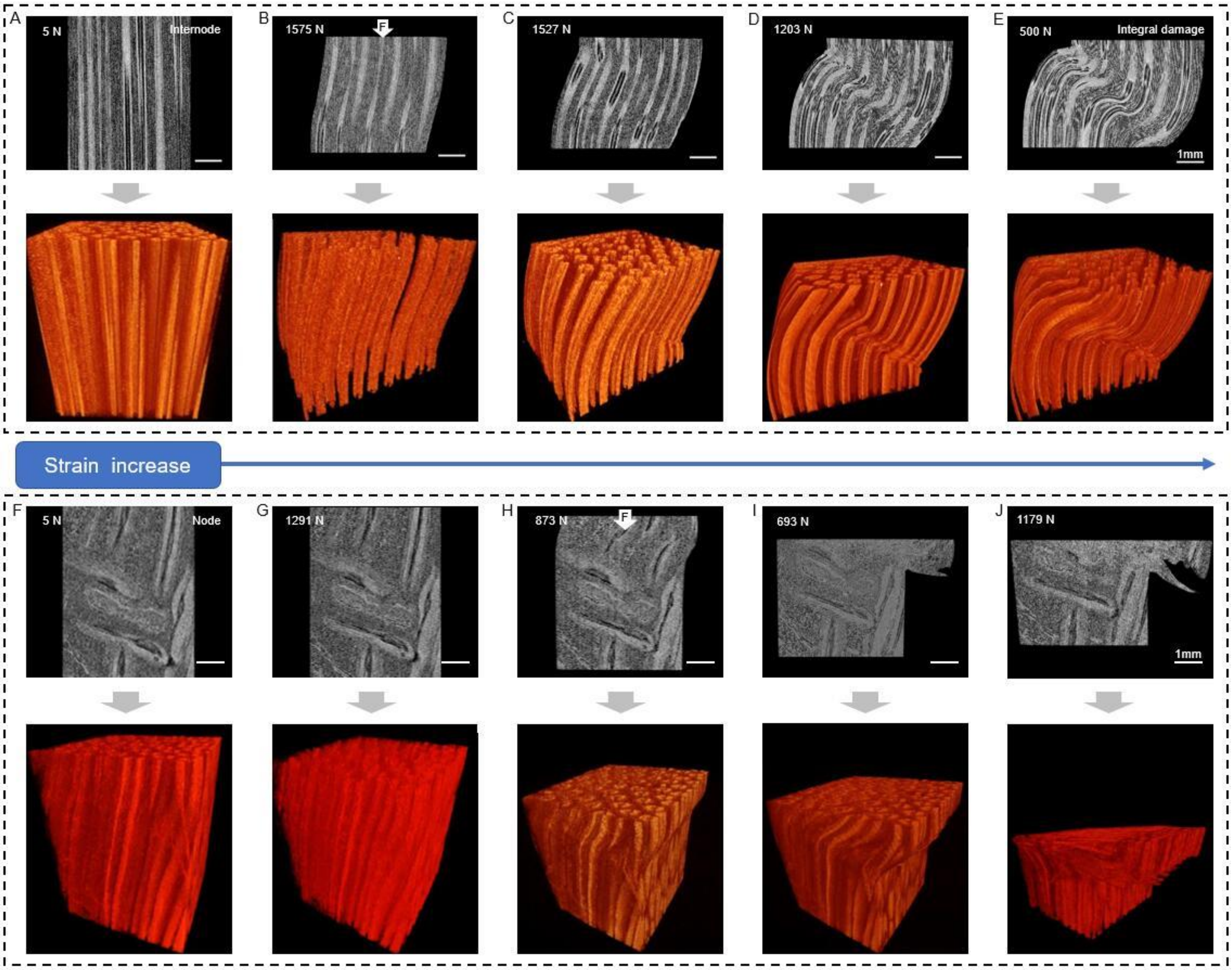

Fig. S19.
Slice projections and reconstructed 3D configurations of the structural evolution of the internode culm (**A**-**E**) and node culm (**F**-**J**) under in-situ compression load conditions.

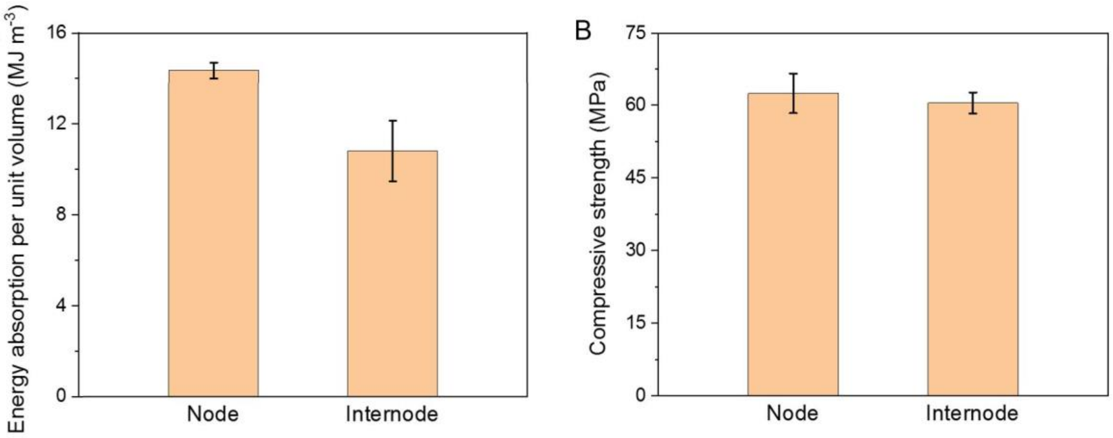

Fig. S20.
Energy absorption and compressive strength of node and internode culms.

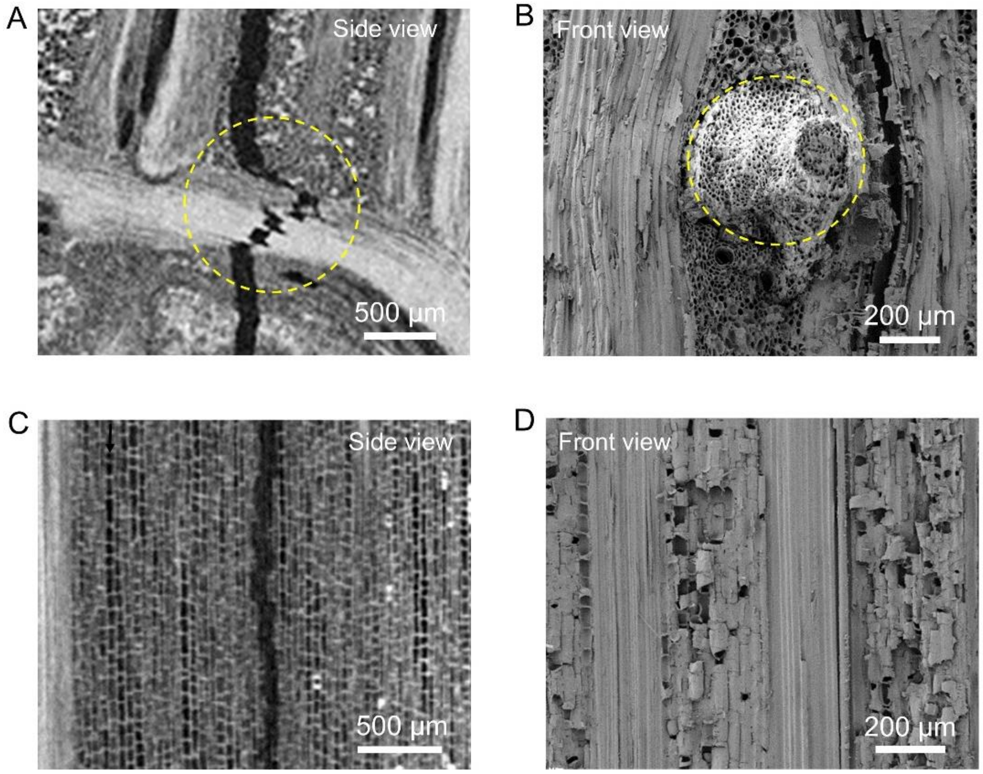

Fig. S21.
Micro-CT (**A**,**C**) and SEM (**B**,**D**) images of the node (**A**-**B**) and internode (**C**-**D**) culms under splitting load. The yellow circles show that the TVB can induce crack deflection. The TVB exhibits a rough fracture cross-section, showing the sliding and pull-out of the constituent microfibers and nanofibers.

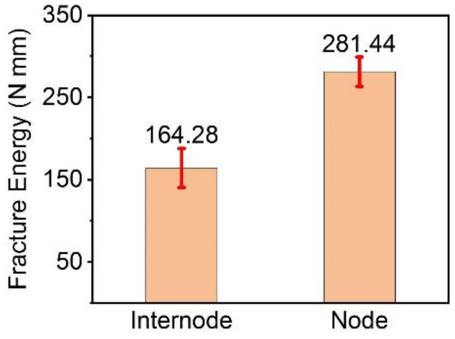

Fig. S22.
Fracture energies of internode and node culms after splitting tests.

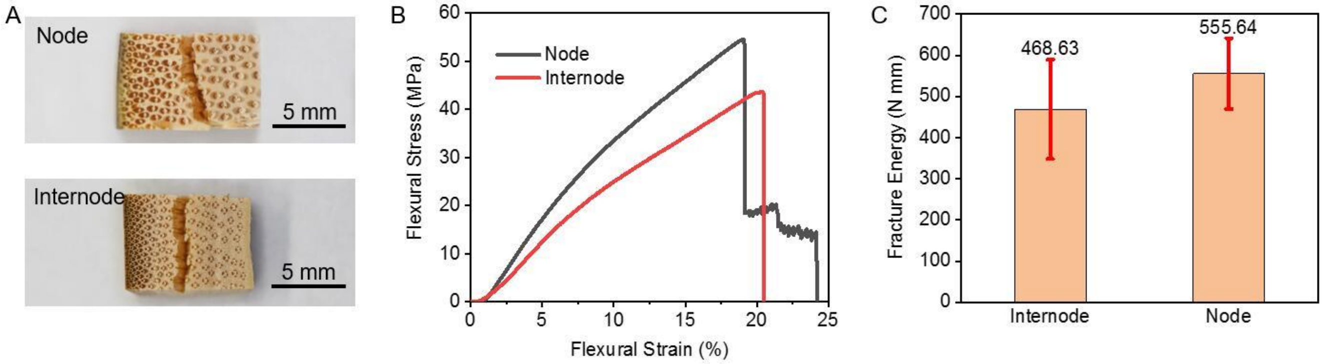

Fig. S23.
Three-point bending tests of node and internode culms. (**A**) Real specimens after tests, load direction is parallel to our view, from the inside (top-loading direction) to the outside (bottom-loading direction). (**B**-**C**) Stress-strain curves and fracture energies of them.

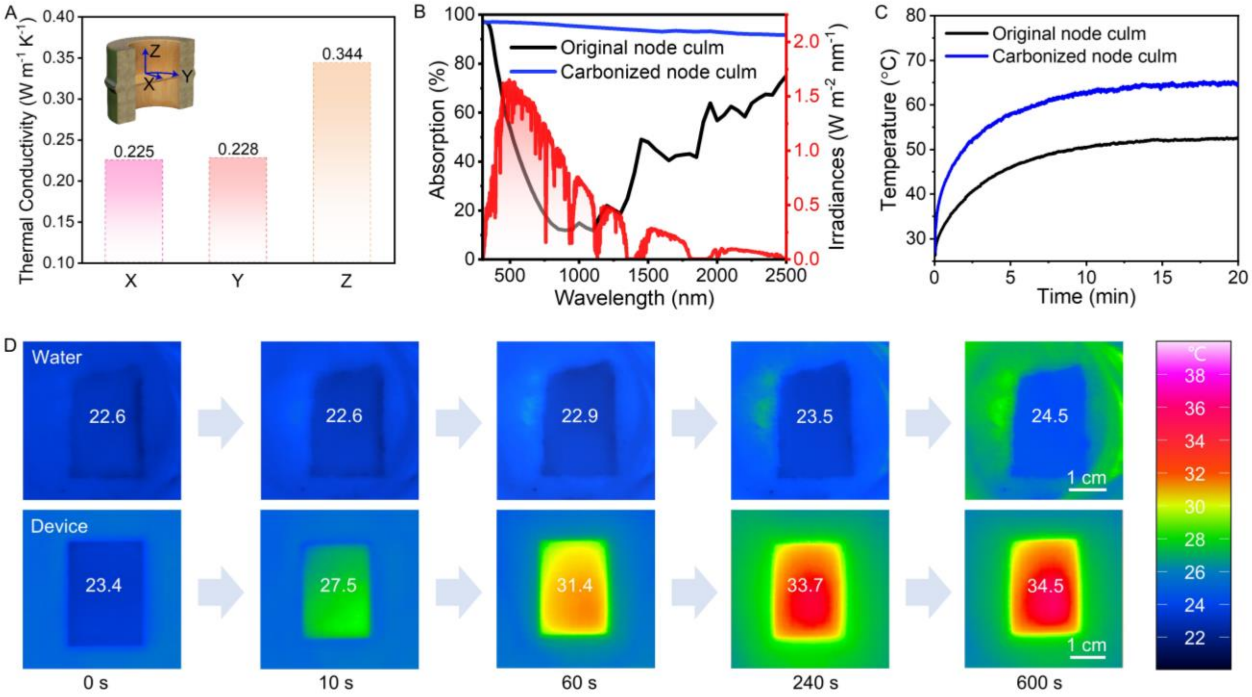

Fig. S24.

Performance of node culm-based solar thermal water evaporation device. (**A**) Thermal conductivities of node culm with different directions (X, radial direction; Y, tangential direction; Z, axial direction), showing anisotropy. (**B**) Total optical absorption of the original and carbonized node culms in the wavelength range from 250 nm to 2500 nm. The carbonized surface exhibits high light absorbance (>90%). The inserted red background represents a standard AM 1.5G solar spectrum. (**C**) Equilibrium temperature curves of the original and carbonized node culms under 1 sun illumination. The carbonized surface can quickly rise to about 65 ℃. (**D**) Thermal IR images of the device during operation.

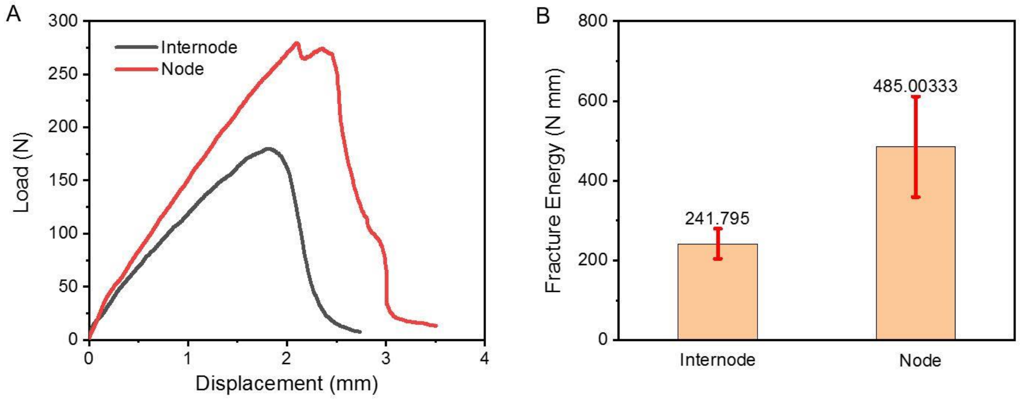

Fig. S25.
Splitting tests of the wet internode and node culms. Load-displacement curves (**A**) and fracture energies (**B**) of them.

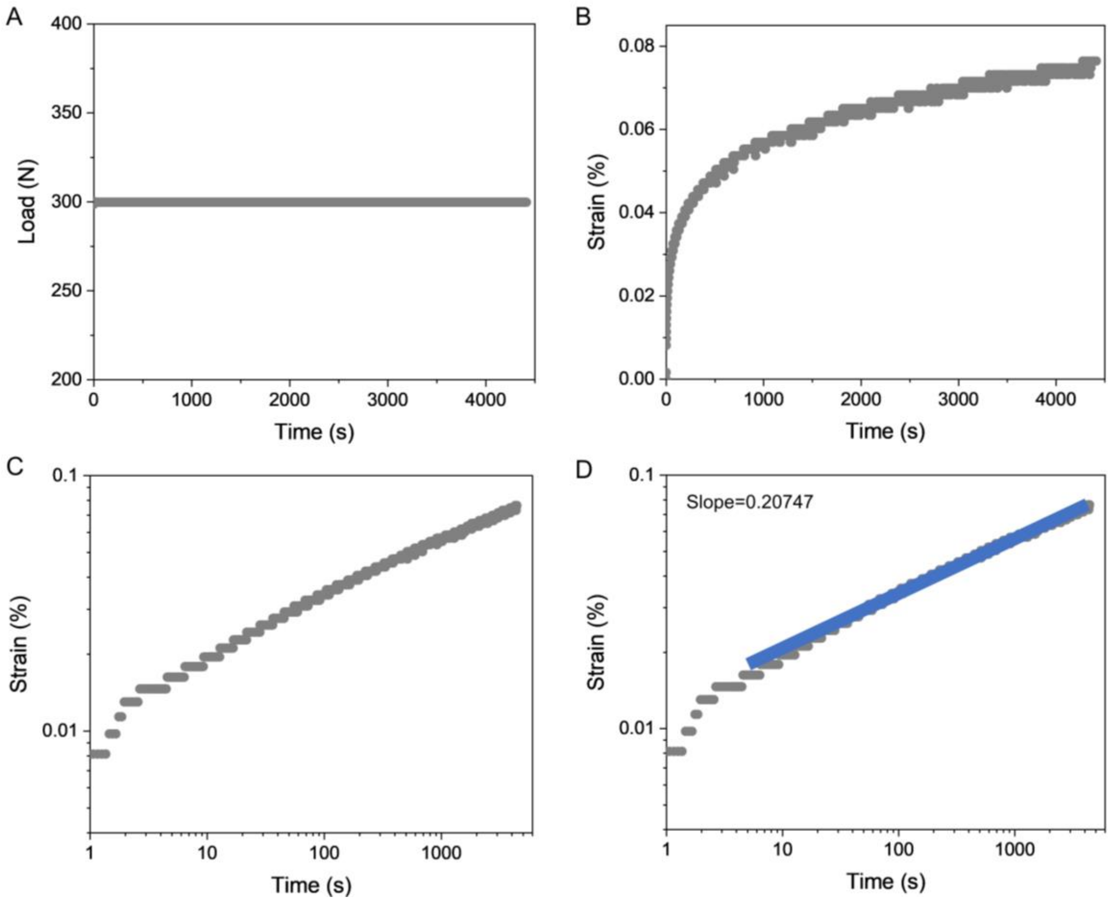

Fig. S26.
Creep test of the bamboo node. (**A**-**B**) Load-time curve and strain-time curve acquired during creep testing. (**C**) Logarithmic strain-time curve. (**D**) Logarithmic strain-time curve and the fitted curve (blue curve).

Table S1.
Comparison between the microfiber length obtained in this work and the data reported in the literatures.

-	Microfiber length (mm)	References
D. giganteus<br>B. sinospinosa<br>D. farinosus<br>B. rigida<br>B. pervariadilis Grandis nin	2.546<br>1.686<br>2.259<br>1.902<br>1.672	3
Dwarf Shengyin<br>Wild moso bamboo	0.78<br>1.388	4
G. brang<br>G. levis<br>G. scortechinii<br>G. wrayi	1.91<br>2.04<br>1.745<br>1.799	5
Phyllostachys edulis<br>Diaphragm<br>Inner culm<br>Middle culm<br>Outer culm	 0.296±0.086<br>0.588±0.286<br>0.67±0.366<br>0.949±0.313	This work

Table S2.
Comparison between the microfiber diameter obtained in this work and the data reported in the literatures.

-	Microfiber diameter (μm)	References
D. giganteus<br>B. sinospinosa<br>D. farinosus<br>B. rigida<br>B. pervariadilis Grandis nin	19.043<br>20.999<br>13.963<br>20.271<br>15.364	3
Dwarf Shengyin<br>Wild moso bamboo	19.404<br>15.026	4
G. brang<br>G. levis<br>G. scortechinii<br>G. wrayi	22.75<br>22.67<br>17.26<br>17.86	5
Phyllostachys edulis<br>Diaphragm<br>Inner culm<br>Middle culm<br>Outer culm	 20.2439±4.37482<br>15.51163±5.43521<br>14.20635±3.33679<br>11.03279±2.42189	This work

Table S3.
Comparison between the mechanical data obtained in this work and the data reported in the literatures.

Bamboos	Compressive strength (MPa)	References
Phyllostachys pubescens:<br>Internode<br>Node	 47.51±7.66<br>48.34±6.77	6
Moso bamboo<br>Internode<br>Node	 56.4±4.97<br>59.8±5.65	7
Moso bamboo	69.1	8
Mao bamboo<br>Kao bamboo	47-62.8<br>37.7-62	9
P. edulis<br>P. bambusoides<br>P. nigra<br>P. meyeri<br>B. stenostachya	48.1<br>59.3<br>45.2<br>55.8<br>46	10
Phyllostachya pubescens (Mao Jue)<br>Bambusa pervariabilis (Kao Jue)	78 dry, 29 wet<br>53 dry, 23 wet	11
Gigantochloa atroviolacea bamboo<br>Internode<br>Node	 50.5<br>52.27	12
Bambusa blumeana<br>Bambusa vulgaris<br>Dendrocalamus asper<br>Gigantochloa scortechinii<br>Gigantochloa levis<br>Balanocarpus hemii<br>Koompasia malaccensis	24<br>25.3<br>31.5<br>27<br>40<br>69<br>54.7	13
Bamboo<br>Internode<br>Node	 50-70<br>45-60	14
Bamboos	53.5-67	15
Phyllostachys edulis<br>Internode<br>Node	 60.47±2.17<br>62.43±4.08	This work

Table S4.

The geometry and materials parameters of the models in the first group.

	Geometry parameters		Materials parameters	
Longitudinal fiber	Width (mm)	1	E_{11} (GPa)	200
			$E_{22}=E_{33}$ (GPa)	20
	Length (mm)	10	$G_{12}=G_{23}=G_{31}$ (GPa)	1
			ν	0.3
Horizontal fiber	Diameter (mm)	0.8	E (GPa)	15
			ν	0.3

Table S5.

The geometry and materials parameters of the models in the second group.

<table>
<tr><td colspan="4">Geometry parameters</td><td colspan="3">Materials parameters</td></tr>
<tr><td rowspan="2">A quarter of the model</td><td>Diameter (mm)</td><td colspan="2">180</td><td rowspan="2">Wall</td><td>E (GPa)</td><td>200</td></tr>
<tr><td>Wall thickness (mm)</td><td colspan="2">30</td><td>ν</td><td>0.3</td></tr>
<tr><td rowspan="5">Fiber along radius (Model#1,3)</td><td>Width (mm)</td><td colspan="2">1</td><td rowspan="4">Fiber</td><td>E_{11} (GPa)</td><td>150</td></tr>
<tr><td rowspan="2">Length (mm)</td><td>Model#1</td><td>15</td><td>$E_{22}=E_{33}$ (GPa)</td><td>15</td></tr>
<tr><td>Model#3</td><td>60</td><td>$G_{12}=G_{23}=G_{31}$ (GPa)</td><td>6</td></tr>
<tr><td rowspan="2">Amount</td><td>Model#1</td><td>19</td><td>ν</td><td>0.3</td></tr>
<tr><td>Model#3</td><td>5</td><td rowspan="2">Matrix</td><td>E (GPa)</td><td>0.6</td></tr>
<tr><td rowspan="3">Fiber along circumference (Model#2)</td><td>Width (mm)</td><td colspan="2">0.8</td><td>ν</td><td>0.3</td></tr>
<tr><td>Length (mm)</td><td colspan="2">30</td><td rowspan="2">Diaphragm</td><td>E (GPa)</td><td>1</td></tr>
<tr><td>Amount</td><td colspan="2">12</td><td>ν</td><td>0.3</td></tr>
</table>

Movie S1. Reconstructed 3D configuration of the part node.

Movie S2. Reconstructed 3D configuration of the part node culm (top view).

Movie S3. Reconstructed 3D configuration of the part node culm (front view).

Movie S4. Continuous 2D slice projections of the node culm (side view).

Movie S5. Reconstructed 3D configuration of the part node culm highlighting fibrous VBs.

Movie S6. Reconstructed 3D configuration of the node culm-diaphragm transition zone highlighting fibrous VBs.

Movie S7. Continuous 2D slice projections of the part diaphragm and part node culm (front view, from the diaphragm to culm).

Movie S8. Reconstructed 3D crack of the node culm-related arched specimen after compression test.

Movie S9. Reconstructed 3D crack of the node culm after splitting test.

References

1. Cui, J., Qin, Z., Masic, A., & Buehler, M. J. Multiscale structural insights of load bearing bamboo: A computational modeling approach. Journal of the Mechanical Behavior of Biomedical Materials. 2020, 107, 103743.
2. Tan, T., Rahbar, N., Allameh, S. M., Kwofie, S., Dissmore, D., Ghavami, K., & Soboyejo, W. O. Mechanical properties of functionally graded hierarchical bamboo structures. Acta Biomaterialia, 2011, 7, 3796-3803.
3. Fei, B., Gao, Z., Wang, J., & Liu, Z. Biological, anatomical, and chemical characteristics of bamboo. Secondary xylem biology. Academic Press, 2016, 283-306.
4. Wang, T., Liu, L., Wang, X., Liang, L., Yue, J., & Li, L. Comparative analyses of anatomical structure, phytohormone levels, and gene expression profiles reveal potential dwarfing mechanisms in Shengyin bamboo (Phyllostachys edulis f. tubaeformis). International Journal of Molecular Sciences, 2018, 19(6), 1697.
5. Mustafa, M. T., Wahab, R., Sudin, M., Khalid, I., & Kamal, N. A. M. Anatomical properties and microstructures features of four cultivated bamboo Gigantochloa species. Journal of Asian Scientific Research, 2011, 1(7), 328-339.
6. Guan, X., Yin, H., Chen, B., Zhu, Y., Liu, X., & Lin, J. The Effect of Microstructure on Mechanical Properties of Phyllostachys pubescens. BioResources, 2020, 15(1), 1430-1444.
7. Shao, Z. P., Zhou, L., Liu, Y. M., Wu, Z. M., & Arnaud, C. Differences in structure and strength between internode and node sections of moso bamboo. Journal of Tropical Forest Science, 2010, 22, 133-138.
8. Dixon, P. G., & Gibson, L. J. The structure and mechanics of Moso bamboo material. Journal of the Royal Society Interface, 2014, 11(99), 20140321.
9. Lo, T. Y., Cui, H. Z., & Leung, H. C. The effect of fiber density on strength capacity of bamboo. Materials Letters, 2004, 58(21), 2595-2598.
10. Akinbade, Y., Harries, K. A., Flower, C. V., Nettleship, I., Papadopoulos, C., & Platt, S. Through-culm wall mechanical behaviour of bamboo. Construction and Building Materials, 2019, 216, 485-495.
11. Yu, W. K., Chung, K. F., & Chan, S. L. Column buckling of structural bamboo. Engineering Structures, 2003, 25(6), 755-768.
12. Oka, G. M., Triwiyono, A., Awaludin, A., & Siswosukarto, S. Effects of node, internode and height position on the mechanical properties of Gigantochloa atroviolacea bamboo. Procedia Engineering, 2014, 95, 31-37.
13. Chaowana, P. Bamboo: an alternative raw material for wood and wood-based composites. Journal of Materials Science Research, 2013, 2(2), 90.
14. Wakchaure, M. R., and S. Y. Kute. Effect of moisture content on physical and mechanical properties of bamboo. Asian Journal of Civil Engineering (Building and Housing), 2012, 13(6), 753-763.

15. Meng, X., Sun, H., Cao, Y., & Feng, P. Experimental Study on Uniaxial Compression of Bamboo Nodes Using 3D Scanning Technique. MATEC Web of Conferences. EDP Sciences, 2019, 275, 01022.
